# Diel Bacterioplankton Community Dynamics Under Contrasting Light Regimes

**DOI:** 10.1111/1758-2229.70099

**Published:** 2025-05-08

**Authors:** Sofia Papadopoulou, Annika Linkhorst, John Paul Balmonte, Bianka Csitári, Tamás Felföldi, Zsuzsanna Márton, Maliheh Mershad, Attila Szabó, Anders Torstensson, Stefan Bertilsson, Anna J. Székely

**Affiliations:** ^1^ Department of Ecology and Genetics/Limnology Uppsala University Uppsala Sweden; ^2^ Department of Environmental Radioactivity and Monitoring Federal Institute of Hydrology (BfG) Koblenz Germany; ^3^ Lehigh Oceans Research Center Lehigh University Bethlehem Pennsylvania USA; ^4^ Department of Microbiology ELTE Eötvös Loránd University Budapest Hungary; ^5^ Department of Microbiology, Tumor and Cell Biology Karolinska Institute Stockholm Sweden; ^6^ Institute of Aquatic Ecology HUN‐REN Centre for Ecological Research Budapest Hungary; ^7^ Department of Aquatic Sciences and Assessment, Science for Life Laboratory Swedish University of Agricultural Sciences Uppsala Sweden; ^8^ Department of Aquatic Sciences and Assessment Swedish University of Agricultural Sciences Uppsala Sweden; ^9^ Swedish Meteorological and Hydrological Institute Community Planning Services ‐ Oceanography Västra Frölunda Sweden

**Keywords:** 16S rRNA, bacterioplankton, diel cycle, freshwater lake, methane, peat bog

## Abstract

In the Boreal region, extreme seasonal variations in day–night length expose communities to dynamic light and temperature fluctuations. Freshwater bacterioplankton, representing key ecosystem components, faces climate‐driven shifts; yet the fixed day‐length patterns determined by latitude underscore the importance of studying light's role in predicting ecosystem responses. We investigated bacterial community composition in a brown peat bog and a clear oligotrophic lake across seasons with contrasting light regimes: the summer solstice (> 20 h of daylight) and the autumn equinox (equal day‐night length). Using amplicon sequencing of 16S rRNA transcripts, alongside measurements of physicochemical parameters, organic matter characterisation and dissolved carbon dioxide and methane gas measurements, we found no diel cycling in the lake during either period or in the peat bog near the summer solstice. However, the structure of bacterial peat bog communities exhibited cyclic changes over diel cycles at the autumn equinox. Twelve amplicon sequence variants, including both phototrophic and heterotrophic taxa, increased in abundance at all measured morning sampling times. These findings provide valuable insights into the diel patterns of boreal lentic habitats and their bacterioplankton communities, highlighting the absence of diel fluctuations in some systems and seasons, while revealing cyclic dynamics in others, driven by conditionally rare taxa.

## Graphical abstract

We assessed the diel variability of bacterioplankton communities and influencing parameters in a boreal peat bog and an oligotrophic lake under two different light regimes: more than 20‐h light prevalence in the summer and equal day and night lengths in autumn. Using amplicon sequencing of 16S rRNA transcripts, combined with biogeochemical measurements, we detected a cyclic pattern in community composition for the peat bog over all measured diel cycles in the autumn.

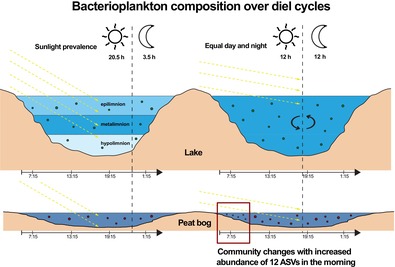

## 1 Introduction

Once every 24 h, the Earth completes a rotation around its axis, subjecting most living organisms to daily fluctuations of light and temperature. Organisms modify their activities over this rhythmic phenomenon, called diel cycle, and a plethora of diel adaptations can be observed across all domains of life (Goodwin and Goodwin 2017). In aquatic ecosystems, planktonic communities show prominent examples of such adaptations, like the variation of photosynthetic carbon dioxide assimilation from phytoplankton (Prezelin 1992) and the vertical migration of zooplankton (Haney 1988). Additionally, changes in the composition of bacterioplankton communities may occur over hourly timescales, with some bacterial taxa being more abundant at particular sampling times (Grubisic et al. 2017; Linz et al. 2020; Shahraki et al. 2020).

Circadian rhythms differ from diel cycles as they are self‐sustaining; they are maintained by endogenous molecular programs, known as circadian clocks, which allow the synchronisation of biological activity with day–night variation (Kondo and Ishiura 2000; Patke et al. 2020; Saini et al. 2019). Circadian clocks are ubiquitous in eukaryotes, including fungi (Baker et al. 2012) and humans (Roenneberg and Merrow 2016), and have more recently been characterised in cyanobacteria (Cohen and Golden 2015; Shalapyonok et al. 1998). There is growing evidence for a widespread existence of circadian clocks even in non‐photosynthetic bacteria and archaea (Eelderink‐Chen et al. 2021; Sartor et al. 2023; Schmelling et al. 2017), which may implicate selective advantages under conditions with balanced day–night length, such as those typical of tropical and temperate regions, or during equinox periods at higher latitudes. However, circadian mechanisms are likely less advantageous under conditions with minimal light fluctuations, such as the extended day or night periods characteristic of high‐latitude regions, or in environments where diel cycling has limited influence, such as the aphotic zone of water bodies. Overall, while the physiological adaptations to day–night patterns start to be understood in prokaryotic strains, the knowledge related to the diel dynamics of natural microbial communities remains limited (Morimoto et al. 2020).

In the boreal region, spanning approximately between 50° and 70° north of the equator, freshwater ecosystems are particularly abundant (Messager et al. 2016) and are characterised by unique environmental conditions, including extreme seasonal variations in daylight cycles. These variations make diel dynamics of boreal bacterioplankton especially intriguing and relevant. The growing period (i.e., when conditions are suitable for the active growth of photosynthetic organisms) typically extends from May to September and is marked by substantial changes in day length (photoperiod). Around the summer solstice (May to July), long‐day photoperiods with extended daylight prevail, while closer to the autumn equinox (August to September), day and night lengths become more balanced. The duration of the photoperiod regulates the growth of some boreal macroorganisms, such as forest trees, ensuring the completion of physiological processes before the frost sets in (Bonan and Shugart 1989). Seasonal changes in day length also determine the optimal time for bird breeding in high‐latitude systems (Sharp 2005). Recently, short‐term dynamics of microbiomes have been explored in biogeochemically distinct systems, as in Linz et al. (2020) who reported similar diel patterns of gene expression across a eutrophic, an oligotrophic and a humic lake. However, the influence of diel cycles remains largely unexplored under seasonally different light regimes. This gap also extends not only to microbial activity but also to diel dynamics of dissolved organic carbon (Gasol et al. 1998; Herndl and Malacic 1987) and chromophoric dissolved organic matter (CDOM; Watras et al. 2015), which are strongly linked to bacterial community composition (Amaral et al. 2016).

Arctic amplification, the accelerated warming of high‐latitude regions (Cohen et al. 2014), and the crucial role of boreal freshwater systems in carbon cycling (Tranvik et al. 2018) highlight the importance of boreal lakes in the context of a changing climate (Leifeld and Menichetti 2018; Yvon‐Durocher et al. 2014). While the seasonal variation in day length typical of this region is not being altered by the changing climate, warming temperatures, shorter winters, and longer ice‐free periods are creating conditions more characteristic of lower latitudes (Sharma et al. 2021). This climate shift, combined with the stable boreal light cycles, will produce a novel set of environmental conditions: a climate resembling that of lower latitudes, but paired with the distinctive seasonal light patterns of the boreal region. It has been shown that such conditions may pose barriers to macroorganisms, such as the poleward distribution of fish populations (Ljungström et al. 2021), and this unique combination of factors could have profound effects also on freshwater ecosystems and their microbial communities. Moreover, environmental changes may alter the light conditions within these systems. For instance, climate change‐driven warming could intensify thermal stratification during the boreal growing period, potentially deepening the photic zone and modifying light availability (Seifert et al. 2023). Furthermore, higher temperatures could influence non‐biological, light‐mediated processes, such as the photodegradation of CDOM, further modulating light conditions in boreal water systems (Porcal et al. 2015). All in all, elucidating the interplay between natural bacterial communities and the seasonally varying boreal light cycles, as well as shedding light on the factors that regulate diel microbial processes, is crucial for understanding the microbial contribution to key functions of these ecosystems and predicting their response to the changing climate.

With this study, we aimed to investigate the influence of diel cycles on the bacterioplankton communities of two boreal water bodies, a humic peat bog and a clearwater oligotrophic lake, and for the first time, compare diel bacterial dynamics in natural aquatic ecosystems during two distinct light regimes: long‐day photoperiod in June, close to the summer solstice when complete darkness was absent, and September, during the autumn equinox, when day and night were of equal length. To capture the dynamic responses of bacterioplankton to diel changes, we assessed the composition of bacterial communities based on their ribosomal RNA (rRNA) pool through amplicon sequencing of 16S rRNA transcripts, as day–night fluctuations may influence microbial activity without a substantial change in community composition (Ruiz‐González et al. 2012). Additionally, recognising the crucial role of boreal freshwater ecosystems in organic matter processing and carbon cycling, we analysed various environmental parameters, characterised the qualitative properties of dissolved organic matter (DOM), and measured carbon dioxide and methane concentrations to explore their link to diel shifts in bacterial taxa. We hypothesised that: (a) the impact of diel cycles on bacterial community structure will have more pronounced effects in the clearwater lake compared to the peat bog, where the prevalence of light‐absorbing organic matter is expected to mitigate the influence of seasonally varied light fluctuation; (b) during the long‐day photoperiod in June, diel oscillations in community composition will be less pronounced compared to September, when day and night lengths are balanced; (c) Community structure in the clearwater lake will exhibit greater variation in the surface water compared to the bacterioplankton communities deeper in the photic zone, where light availability, and consequently, the impact of light fluctuations, is reduced. Our findings revealed a diel pattern in the bacterial community of the peat bog only in autumn, which was dominated by a small subset of taxa that included both phototrophic and heterotrophic members of the microbial community.

## 2 Materials and Methods

### 2.1 Diel Sampling

Two oligotrophic water bodies in Jämtland county, Sweden, were selected for this study: Klocktjärnen (hereafter called ‘lake Klocka’) and an unnamed humic peat bog. Both had catchments dominated by forests and wetlands (Figure S1). Two sampling campaigns were conducted in 2019 during periods of contrasting light regimes. The first sampling was conducted in June (23–27/06), when daylight prevailed for 20.5 h between sunrise and sunset, and the remaining 3.5 h of the day were twilight conditions (Figure S2). The second campaign was in September (19–23/09) when the day and night were of equal duration, that is, 12 h. For each sampling campaign, water samples were collected at 6‐h intervals over four diel cycles with 15 time points in June and 17 time points in September. The sampling times were the same for both water bodies and the time points corresponded to the chronological order of collected samples (Table 1). The particular time of sampling (19:15, 01:15, 07:15 and 13:15) was determined by the local solar noon (13:15), the time when the Sun reached its highest position (Figure S3).

**TABLE 1:** Sampling times and their corresponding time points (1–17) across the four diel cycles during each sampling campaign.

Hour	01:15	07:15	13:15	19:15		01:15	07:15	13:15	19:15
Light conditions	Twilight	Daylight	Solar noon	Daylight		Night	Sunrise	Solar noon	Sunset
23‐June				1	19‐September				1
24‐June	2	3	4	5	20‐September	2	3	4	5
25‐June	6	7	8	9	21‐September	6	7	8	9
26‐June	10	11	12	13	22‐September	10	11	12	13
27‐June	14	15			23‐September	14	15	16	17

*Note:* The time points were consistent across both water bodies and all sampled depths.

Based on light conditions, five sampling depths were selected in lake Klocka (0.2, 1, 2.5, 5.5 and 9 m) to capture microbial communities under contrasting light levels, and water samples were collected from each depth using a Ruttner sampler. In the lake, the sediment located at 10 m depth was not disturbed while taking the deepest samples or the corresponding in situ measurements. Meanwhile, only one depth (0.2 m) was sampled in the shallow peat bog. For both sites, the 0.2 m samples were taken from below the water surface, excluding any surface film. Altogether, 192 samples were collected for each measurement conducted (15 time points in June × (5 lake + 1 peat bog) depths + 17 time points in September × (5 + 1) depths).

### 2.2 Physicochemical Parameters

Temperature and illuminance were continuously recorded (every minute) in the water as well as in the air above the water surface with HOBO data loggers (HOBO Pendant temperature/light 64 K data logger, Onset Computer Corporation). At the same time points and depths as the sample collection, measurements of the following environmental parameters were performed in situ with a portable probe (YSI 6600 V2): water temperature, electrical conductivity, dissolved oxygen (DO) concentration and its saturation (%). We also measured pH from unfiltered water directly after sampling on a mobile pH meter (Metrohm 826 with Aquatrode Plus electrode; calibrated with Merck Certipur buffers).

Total phosphorus (TP), total organic carbon (TOC) and total nitrogen (TN) were measured for the peat bog and the five depths of the lake at time point 1 during the autumn sampling for the general determination of the trophic status of the water bodies. TP concentration was measured photometrically at 882 nm (Lambda 40 UV/Vis spectrophotometer, Perkin Elmer) and in triplicates using the molybdenum‐blue method after oxidative hydrolysis with potassium persulfate in acidic solution at high temperature and pressure in an autoclave (Menzel and Corwin 1965; Murphy and Riley 1958). TOC, measured as non‐purgeable organic carbon, and TN were analysed in triplicates on a Shimadzu TOC‐L/TNM‐L with potassium hydrogen phthalate (KHP) as the standard for TOC and ethylenediaminetetraacetic acid (EDTA) as a secondary standard to check the calibration curve. For TN, calibration was done with potassium nitrate (KNO_3_). The samples were mixed before sample injection.

Information about the weather conditions during the sampling campaigns was collected from the Swedish Meteorological and Hydrological Institute (SMHI). Hourly data for cloud coverage, air temperature, relative humidity, wind speed and wind direction and quarter‐hour data for the precipitation amount were received from the local weather station (i.e., Storlien‐Storvallen) for each diel cycle. Only controlled and approved values by SMHI were considered for the analyses.

### 2.3 Gas Measurements

For all time points and depths, we collected water in 60 mL syringes, in triplicates. Samples were brought back to the laboratory, where the same volume of air was added in the syringes, and samples were shook thoroughly for 1 min, performing the headspace technique (Cole and Caraco 1998). The gas was then injected to an ultraportable greenhouse gas analyser (UGGA, Los Gatos Research) for measurement of the partial pressure of carbon dioxide (pCO_2_) and methane (pCH_4_) concentrations according to Paranaíba et al. (2018).

### 2.4 Chlorophyll *a*

Unfiltered lake water was passed through Whatman GF/F Glass microfiber filters (0.7 μm) until clogging, upon which the filtered volumes were recorded for the 192 filters produced. Filters were stored at −20°C prior to analysis. The extraction and concentration of chlorophyll *a* (chl‐*a*) from the frozen filters were performed by absorbance as in Garcia et al. (2019).

### 2.5 Organic Matter Characterisation

Water was collected in combusted glass vials (4 h, 450°C) and stored at 4°C in the dark until spectroscopic analysis. Absorbance and fluorescence measurements were performed according to Kothawala et al. (2014), with a Lambda 40 UV/Vis spectrophotometer (Perkin Elmer) and a SPEX FluoroMax‐4 spectrofluorometer (Horiba Jobin Yvon) respectively. Excitation emission matrices (EEM) were characterised by parallel factor analysis (PARAFAC) for all samples, using the DOMFluor toolbox (Stedmon and Bro 2008) in MATLAB software (version 7.7.0). The underlying fluorescence components and their corresponding peaks were identified, absorbance at 350 nm (*A*350) was measured, and the fluorescence (FI), freshness (*β*:*α*) and humification (HIX) indices were calculated as described by Kothawala et al. (2012).

### 2.6 Bacterial Community Composition

Microbial cells were concentrated from the water by filtration onto 0.1 μm pore size filters (Supor polyethersulfone membrane, Pall Corporation) shortly after sampling. Filters were transferred to liquid nitrogen until return to the laboratory for long‐term storage at −80°C until nucleic acid extraction. Library preparation involved combined DNA/RNA extraction using the Easy DNA kit (Invitrogen; Székely et al. 2013). For the peat bog extracts, the RNeasy PowerClean Pro Cleanup kit (QIAGEN) was used to eliminate potential inhibitors, such as humic substances, and increase RNA purity. DNase I (Invitrogen) was used to remove DNA from the extracts according to the manufacturer's instructions. All DNase‐treated samples were tested for DNA remains with a 35‐cycle polymerase chain reaction (PCR) test using the same primers as for amplification (see below), and if DNA was detected, this process was repeated.

After a successful DNase treatment, RNA was reverse transcribed into complementary DNA with random hexamer primers, using the RevertAid H Minus First Strand cDNA synthesis kit (Thermo Fisher Scientific), and products were directly used for PCR amplification. PCR products were prepared for amplicon sequencing based on Vass et al. (2020). In a first 20‐cycle PCR, duplicates of each sample were amplified using the Illumina adaptor‐linked primers 341F (5′‐NNNNCCTACGGGNGGCWGCAG‐3′) and 805R (5′‐GACTACNVGGGTATCTAATCC‐3′), which amplified the V3‐V4 hypervariable region of bacterial 16S rRNA. Sample‐specific index sequences (barcodes) were introduced in the subsequent 15‐cycle PCR, ensuring that individual samples would be properly identified after sequencing. After each PCR step, products were cleaned with magnetic beads (MagSi‐NGS^PREP^ Plus, magtivio). DNA was quantified with PicoGreen assays (Quant‐It PicoGreen, Invitrogen) and the pooled library was purified with the QIAquick Gel Extraction kit (QIAGEN). Sequencing was performed on an Illumina MiSeq v3 flow cell (cluster generation and paired‐end sequencing, 300 cycles).

### 2.7 Bioinformatic Community Analysis and Statistics

The DADA2 pipeline (version 1.8; Callahan et al. 2016) in R (version 4.0.0) was used for the construction of the amplicon sequence variant (ASV) table. Taxonomic assignments of the sequences were based on a training set of Silva reference sequences (version 138.1; McLaren and Callahan 2021; Quast et al. 2013). The minimum bootstrap confidence for assigning a taxonomic level was set to 50. The amplicon sequencing data were further processed in R studio (version 2021.09.0), using the phyloseq (version 1.38.0; McMurdie and Holmes 2013) and vegan (version 2.6‐4; Oksanen et al. 2022) packages. For the calculation of alpha and beta diversity, ASV matrices were normalised by rarefying without replacement per site (*rarefy_even_depth* function in phyloseq), resulting in 12,474 reads per peat bog sample, while for the lake or when all communities were compared, 7468 total reads.

The Shannon index (Shannon 1948) was calculated through phyloseq. ANOVA tests (stats package in R; version 4.1.1) were applied to test the effects of month and depth on the alpha diversity index. Post hoc comparisons with the Tukey HSD test (rstatix package; version 0.7.1) served to determine if the mean difference between specific pairs was statistically significant. In case the assumptions of either normality or homogeneity of variances of the residuals, or both, were not fulfilled, even after transforming the dependent variable, an ANOVA with *p* values based on permutation tests followed, using the permuco package (version 1.1.2). The number of permutations used was 100,000 and the test was adjusted with Bonferroni corrections (Dunn 1961, 1959). In case an ANOVA with permutation tests was used, only these results are reported.

ASV counts of the non‐rarefied data were transformed into relative abundance to visualise fluctuations of bacterial community composition across time, at phylum, class, and family taxonomic levels. The relative abundance dataset for both systems was also scanned for ASVs that exhibited their highest relative abundance consistently at a specific sampling time. In that case, the ASV abundances were transformed with the z‐score for visualisation purposes. In addition, the Kendall's Tau correlation coefficient was calculated to analyse the relationship between the relative abundances of these ASVs and the measured environmental factors, using the *corr.test* function from the psych package (version 2.3.12). The resulting correlation coefficients and their respective *p* values were visualised on a heat map. The presence of collinearity among the environmental variables was assessed by calculating a correlation matrix using Kendall's Tau correlation coefficient as above.

For the analysis of beta diversity, microbial communities in the lake and the peat bog, or separately, were visualised for the two sampling periods with a non‐metric multidimensional scaling (NMDS) based on a Bray–Curtis dissimilarity matrix, constructed with the *ordinate* function of the phyloseq package. Permutational multivariate analysis of variance (PERMANOVA) with the *adonis2* vegan function was applied to identify statistically significant (*p* < 0.05) clustering of microbial communities. All statistical beta diversity tests used 999 permutations and Bonferroni correction to identify significant clustering. Differences in community dispersion, measured as the distance from the group centroid, were assessed using the vegan *betadispe*r function. ANOVA tests followed to test for significant differences among group beta dispersions. The environmental variables covarying with bacterioplankton communities were fitted in the ordination, using the *envfit* function from the vegan package and only the significant correlations (*p* < 0.01) were visualised in the different NMDS plots.

## 3 Results

### 3.1 Physicochemical Characteristics and Weather Conditions

Lake Klocka was stratified in June, exhibiting a typical pattern of an oligotrophic lake during summer stratification: temperature decreased with depth, while the concentration of DO increased (Figure S4). The lake was supersaturated with oxygen (> 100% saturation) at all measured depths. The epilimnion extended between 0 and 2.5 m depth, while the metalimnion ranged from 2.5 to 5.5 m, and the deepest sampling depth (9 m) was in the hypolimnion. In contrast, the lake was mixed during the September sampling, as indicated by stable temperature and DO concentrations throughout the water column. The lake was slightly alkaline with a pH just above 8, while the peat bog was highly acidic with a pH below 5 (Table S1).

Nutrient concentrations, measured once during the sampling campaign in September, were low for both water bodies (Tables, S2) and corroborated their classification as oligotrophic systems. The ratio of total nitrogen to total phosphorus reflected a relative phosphorus limitation in both systems, which was higher for the peat bog (Table S2). Temperature and light in the air and water, as measured with HOBO loggers, captured distinct diel fluctuations in both study systems and study periods (Figure S5, Table 2). Minor events of precipitation and high cloud coverage were recorded for both sampling periods (Figure S6).

**TABLE 2:** Location, physical and environmental characteristics for the two study systems.

	Lake Klocka	Peat bog
GPS coordinates (WGS84 dec)	N 63.29883	N 63.307035
	E 12.48838	E 12.483308
Altitude (m)	534.5	532.0
Surface area (m^2^)	196,300	1400
Maximum depth (m)	~10	~0.4
Total phosphorus (μg/L)	2.38	2.36
Total nitrogen (μg/L)	127.1	275.1
Total Organic Carbon (mg/L)	2.2	17.4
Temperature (°C)—June, 0.2 m	15.05 ± 0.68	15.77 ± 1.94
Temperature (°C)—June, 9 m	10.90 ± 0.16	—
Temperature (°C)—September, 0.2 m	10.06 ± 0.28	7.48 ± 1.02
Temperature (°C)—September, 9 m	9.96 ± 0.20	—
Illuminance (lux)—June, 0.2 m	561.89 ± 1187.02	2462.78 ± 3452.58
Illuminance (lux)—June, 9 m	340.85 ± 553.73	—
Illuminance (lux)—September 0.2 m	33.56 ± 71.18	272.26 ± 801.12
Illuminance (lux)—September, 9 m	43.06 ± 88.44	—
Chl‐*a* (μg/L)—June	0.50 ± 0.42	0.35 ± 0.34
Chl‐*a* (μg/L)—September	0.81 ± 0.56	0.11 ± 0.09

*Note:* Nutrient values for the lake represent mean measurements across different depths during the September sampling campaign. Water temperature, water illuminance, and chlorophyll *a* values are presented as mean measurements with the standard deviation (mean ± SD) for both sampling periods.

### 3.2 Chlorophyll *a*

Fluctuations in chl‐*a* concentrations were observed over 6‐h intervals in both systems, but no clear diel pattern emerged (Figure S7). Chl‐*a* concentrations were significantly higher in September in the lake (two‐way ANOVA with permutation tests, *F*_1,148_ = 15.28, *p* < 0.001), while no significant difference was observed between depths (*F*_4,148_ = 0.89, *p* = 0.48). The interaction between sampling month and depth was also significant (*F*_4,148_ = 2.94, *p* = 0.02). The chl‐*a* variation was more pronounced in September (Table 2) with lake Klocka reaching the highest chl‐*a* concentration of 2.35 μg/L at time point 12. In contrast, in the peat bog, chl‐*a* concentrations were significantly lower in September (ANOVA, *F*_1,29_ = 7.42, *p* = 0.01), while the variation across the sampling period was more pronounced in June.

### 3.3 Dissolved Organic Matter Composition

Five fluorescence PARAFAC components were identified for each site: Three humic‐like components, similar to peaks A, C and M, which were largely of terrestrial origin, and two protein‐like components, similar to peaks T and B, containing organic structures that are mainly of microbial or phytoplankton origin (Coble et al. 1998; Coble et al. 1990; Fellman et al. 2010; Parlanti et al. 2000, Figure S8). Fluorescence metrics are reported for each sampling period (Table 3). Peat bog samples had a terrestrially derived signature as the FI was close to 1.2, and the FI did not change substantially between June and September, given that a difference less than 0.1 is considered negligible (McKnight et al. 2001). FI in lake Klocka was between 1.2 and 1.8, and this value remained stable during the two sampling periods. The *β*:*α* index was less than 1 for both sites, suggesting that DOM was predominantly decomposed rather than recently derived (Fellman et al. 2010). Higher values of HIX showed that the peat bog contained more humic substances than the lake (Fellman et al. 2010), as expected.

**TABLE 3:** Spectral metrics (mean ± SD) in the peat bog and lake Klocka for both sampling periods.

Site	FI	*β*:*α*	ΗΙΧ	*A*350 (m^−1^)
June				
Peat bog	1.27 ± 0.08	0.41 ± 0.08	13.77 ± 3.58	45.24 ± 1.39
Lake Klocka	1.50 ± 0.05	0.81 ± 0.18	1.69 ± 0.33	1.32 ± 0.37
September				
Peat bog	1.22 ± 0.00	0.34 ± 0.01	18.13 ± 1.38	56.77 ± 1.70
Lake Klocka	1.50 ± 0.04	0.77 ± 0.05	1.57 ± 0.22	1.01 ± 0.46

### 3.4 Partial Pressure of Carbon Dioxide and Methane

**FIGURE 1:**
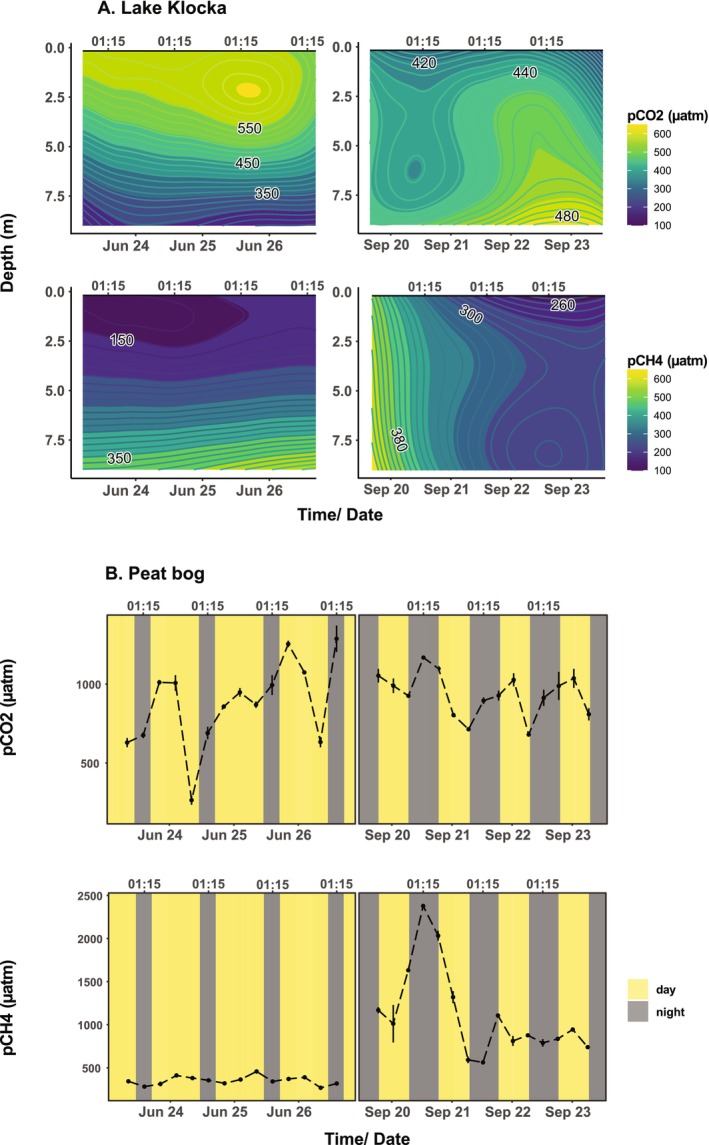
(A) Contour plots for carbon dioxide (CO_2_; upper plots) and methane (CH_4_; lower plots) partial pressure in lake Klocka in June (‘Jun’) and September (‘Sep’), across sampling time and depths. A LOESS (locally estimated scatterplot smoothing) model was fitted in the *z*‐axis (gas partial pressure) to generate the plots. (B) Partial pressure of CO_2_ (upper plots) and CH_4_ (lower plots) over diel cycles in the peat bog. Measurements were done in triplicates, and vertical lines indicate the standard deviation from the mean values.

For the June sampling, pCO_2_ in stratified lake Klocka was higher in the epilimnion and decreased across the meta and hypolimnion (Figure 1A). The opposite pattern was observed for pCH_4_. In September, the lake was mixed, so that for both gases, the partial pressure had similar levels at all measured depths. In the peat bog, pCO_2_ fluctuated over diel cycles during both sampling campaigns with the lowest values usually measured at the last sampling of the day (19:15; Figure 1B). While pCH_4_ remained at stable levels during the June campaign in both systems, it decreased gradually over time in the lake. In the peat bog, an episodic increase in pCH_4_ (2378.76 μatm) coincided with a minor precipitation event on the 20th of September (Figure S6, 1B).

### 3.5 Bacterial Diversity and Community Composition

Details about data output after processing through the DADA2 pipeline and rarefaction curves (Figure S9) can be found in the Supporting Information. Out of the 192 samples processed with DADA2, three samples (two from the lake and one from the peat bog) had less than 7000 reads and were therefore excluded from further analysis.

Alpha diversity, calculated as the Shannon diversity for the lake, was significantly higher in September (two‐way ANOVA with permutation tests, *F*_4,148_ = 11.02, *p* < 0.01), but there were no statistically significant differences across depths (*F*_4,148_ = 1.13, *p* > 0.05), or any significant interaction between depth and sampling period (*F*_4,148_ = 0.93, *p* > 0.05; Figure S10). There were no significant differences between pairs of depths after conducting pairwise comparisons with the Tukey test. The peat bog exhibited significantly lower species richness in September than in June (ANOVA, *F*_1,29_ = 21.95, *p* < 0.001; Figure S11).

**FIGURE 2:**
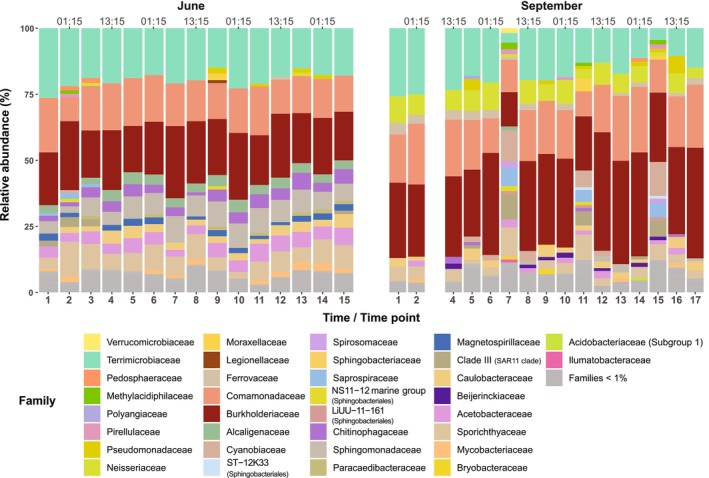
Relative abundance of bacterial families in the peat bog across both sampling periods. Bars represent individual samples and each colour illustrates a different bacterial family. Taxa with relative abundances less than 1% in a sample are grouped together. ASVs with unassigned taxonomy at the family level (bootstrap less than 50) are not included in this plot, but can be found in Figure S13. The sample at time point 3 in September had zero reads and thus, no data are presented.

The taxonomic composition of the bacterioplankton community of the two systems comprised phyla commonly found in aquatic environments, such as Proteobacteria, Actinobacteriota, Bacteroidota, Cyanobacteria and Verrucomicrobiota (Newton et al. 2011). At the class level, the variation of the detected taxa across time points in the lake did not exhibit a diel pattern in either of the two light regimes studied and, overall, the community composition remained stable (Figure S12). Similarly, the bacterial community composition in the peat bog did not vary over time for the entire June sampling. Interestingly, for the September sampling period, the stability of the bacterioplankton community composition was disrupted by three samples collected at 07:15 (i.e., time points 7, 11 and 15, hereafter called “morning communities”) and this was observed already at the class and family level (Figures 2 and S13). The structure of these communities clearly differed from all the other time points and was also highly similar among themselves, indicating a cyclic pattern.

**FIGURE 3:**
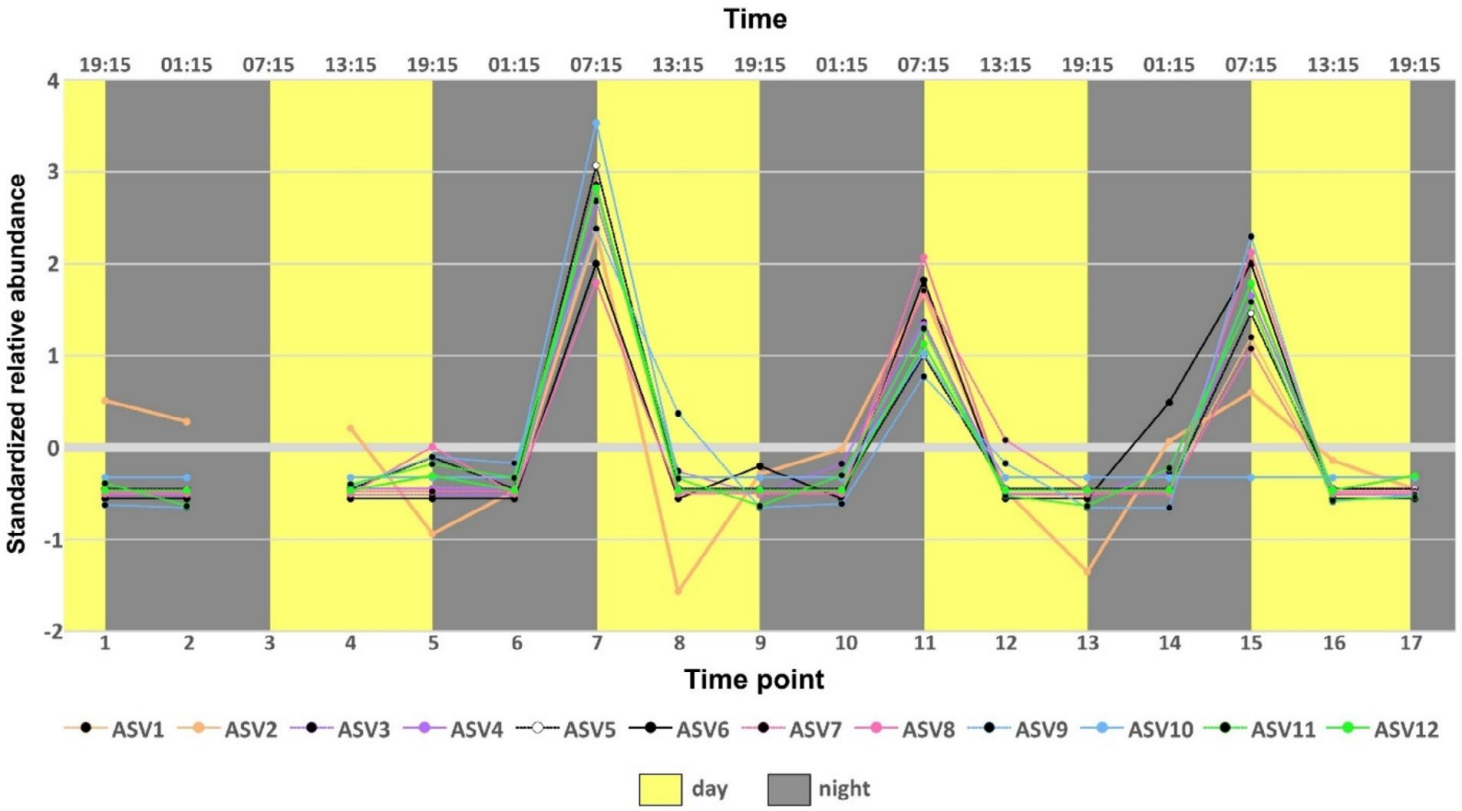
Relative abundance, transformed as the *z*‐score, for the 12 early riser ASVs that exhibited constantly their highest relative abundances at 07:15 in the peat bog during the September sampling period. The sample at time point 3 in September had zero reads and thus, no data are presented.

At a higher resolution, we found 12 ASVs from the peat bog morning communities that exhibited their highest relative abundances at 07:15 in all three fully measured diel cycles (‘early risers’; Table 4; Figures 3 and S14). The early riser ASVs belonged to five different phyla, and five of them could be classified down to genus level. Eleven of these early riser ASVs were classified as members of heterotrophic bacterial taxa, while one, ASV9, was classified as *Cyanobium* PCC‐6307. Apart from ASV9, two further ASVs from Cyanobacteria were identified in the September peat bog samples: *Aphanizomenon* NIES81 (Family Nostocales) at two time points, and *Synechococcus* MBIC10613 (Family Cyanobiaceae) at three time points across different hours, each at low abundance below 1%. In contrast, no distinct diel pattern could be identified for the early riser taxa in the June dataset (Figure S15).

**TABLE 4:** Taxonomic classification of the 12 early riser ASVs in the peat bog community that, in all diel cycles examined in September, consistently exhibited their highest relative abundance at 07:15 (time points 7, 11 and 15).

Taxonomy					ASV code
Phylum	Class	Order	Family	Genus	
Actinobacteriota	Acidimicrobiia	Microtrichales	Ilumatobacteraceae	CL500‐29 marine group	ASV1
	Actinobacteria	Frankiales	Sporichthyaceae	hgcI clade	ASV2
Bacteroidota	Bacteroidia	Chitinophagales	Saprospiraceae	*Candidatus* Aquirestis	ASV3
		Cytophagales	Microscillaceae	*NA*	ASV4
		Flavobacteriales	NS9 marine group	*NA*	ASV5
		Sphingobacteriales	LiUU‐11‐161	*NA*	ASV6
			NS11‐12 marine group	*NA*	ASV7
			ST‐12 K33	*NA*	ASV8
Cyanobacteria	Cyanobacteriia	Synechococcales	Cyanobiaceae	*Cyanobium* PCC‐6307	ASV9
Proteobacteria	Alphaproteobacteria	Caulobacterales	Hyphomonadaceae	UKL13‐1	ASV10
		SAR11 clade	Clade III	*NA*	ASV11
Verrucomicrobiota	Verrucomicrobiae	Methylacidiphilales	Methylacidiphilaceae	*NA*	ASV12

**FIGURE 4:**
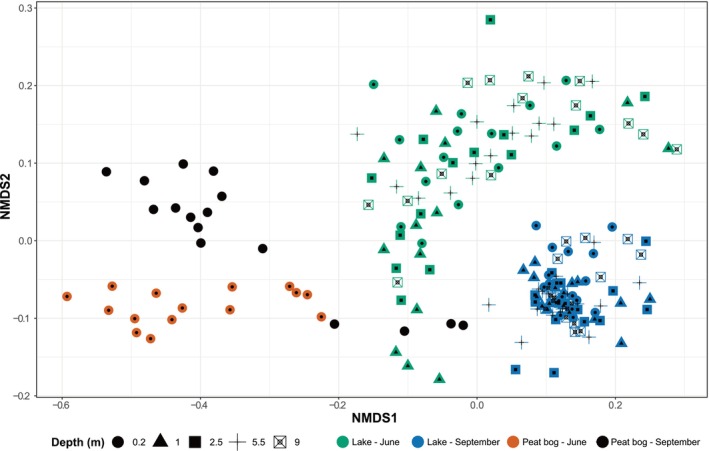
NMDS plot of bacterial communities in the peat bog and lake Klocka (ASV level, stress = 0.196), calculated using a Bray–Curtis dissimilarity matrix for the sampling periods in June and September. Symbols represent individual samples (189 in total), they are coloured according to the combination of site and month and shaped based on depth.

**FIGURE 5:**
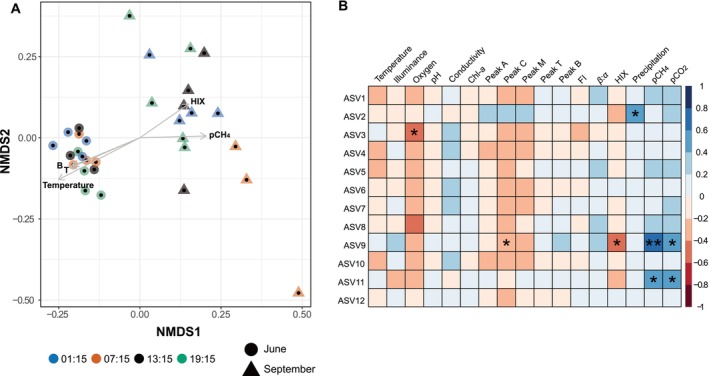
(A) NMDS plot of bacterial communities in the peat bog (ASV level, stress = 0.160), calculated using a Bray–Curtis dissimilarity matrix, for the sampling periods in June and September. Symbols represent individual samples (31 in total) and they are coloured according to sampling time and shaped based on month. Arrows represent significant (*p* < 0.01) correlations of environmental variables with the structure of bacterioplankton communities. The arrows length is proportional to the correlation between ordination and environmental variable. ‘T’ and ‘B’ stand for peaks T and B, respectively. ‘Temperature’ stands for water temperature, ‘HIX’ for the humification index and ‘pCH_4_’ for the partial pressure of methane. (B) Heat map of Kendall's Tau correlation coefficients between the early riser ASVs (rows) and the measured environmental factors (columns). The numerical scale indicates the coefficient, while the colour scale shows the degree of correlation (red for negative correlations; blue for positive correlations). Significance levels are marked with the following asterisks: *0.01 < *p* ≤ 0.05, **0.001 < *p* ≤ 0.01. ‘Oxygen’ stands for the dissolved oxygen concentration in the water, ‘Chl‐*a*’ for chlorophyll *a*, ‘FI’ for the fluorescence index, ‘*β*:*α*’ for the freshness index, ‘HIX’ for the humification index and, ‘pCH_4_’ and ‘pCO_2_’ for the partial pressure of methane and carbon dioxide, respectively.

An NMDS plot for all bacterioplankton communities included in the study revealed separation based on site and sampling period (Figure 4, Figure S16). PERMANOVA (Table S3) confirmed these differences in community structure between sites (*F*_1,187_ = 3.36, *p* = 0.001), months (*F*_1,187_ = 3.60, *p* = 0.001) and the interaction of these two factors (*F*_1,187_ = 1.76, *p* = 0.001). Bacterial communities in the mixed lake in September were clustered significantly closer in the ordination space compared to the stratified communities in June (dispersion: *F*_1,156_ = 97.13, *p* < 0.001). However, there was no obvious clustering with depth even for the stratified summer communities (Figure 4). No visual clustering or significant difference could be detected according to the time of sampling for the lake samples at either of the sampling occasions (Figure S16). The peat bog communities of June and September also presented a clear and significant season‐based separation (PERMANOVA: *F*_1,29_ = 1.49, *p* = 0.001; Figure 4). However, opposite to the lake samples, the peat bog communities clustered closer in June than in September (dispersion: *F*_1,29_ = 8.19, *p* < 0.01). Yet, despite the slight visual separation of the morning communities on the NMDS plot (Figure 5A), there was no significant clustering according to the time of sampling.

Five measured environmental variables showed significant correlations (*p* < 0.01) with the peat bog bacterioplankton community structure. These parameters were the peaks of the protein‐like components (peaks T and B), as well as water temperature, which pointed towards the ordination space of summer peat bog communities. HIX and pCH_4_ featured more strongly in fall communities. According to the Kendall's Tau correlation coefficient, the relative abundance of the early riser ASVs from Figure 3 correlated generally negatively with dissolved oxygen concentration and, to a lesser extent, with humic‐like peaks C and M, as well as the HIX. Meanwhile, they positively correlated with pCO_2_ and pCH_4_. However, these correlations were not statistically significant for most taxa (Figure 5B). In addition, variables that exhibited significant correlations (*p* ≤ 0.05) with others (collinearity) were the following: conductivity, precipitation, four of the fluorescence component peaks (peaks A, M, T and B), *β*:*α* index, HIX, and pCO_2_. Exceptions to this general pattern of environmental correlations of the early riser ASVs are ASV2, which exhibited an opposite correlation with peaks C and M, and a significant positive correlation with precipitation; ASV11, which showed no correlation with peaks C and M; and ASV12, which exhibited a strong correlation only for peak C. No consistent correlation pattern was found for other measured parameters, such as water temperature and illuminance.

## 4 Discussion

In the present study, we assessed diel trends in the composition of bacterioplankton communities in two contrasting boreal waterbodies. To the best of our knowledge, this is one of the few studies attempting to capture the variation of freshwater bacterial communities over highly resolved diel timescales and the first to compare such patterns across two periods with contrasting light regimes. Using amplicon sequencing of the 16S rRNA transcripts and accounting for various environmental parameters, our results add to previous findings on short‐term variation of microbial communities in the boreal region. In the study, we uncovered an obvious cyclic pattern in the composition of a bacterial peat bog community that extended over the full diel cycles studied during a period characterised by an equal day‐ and night‐time length.

### 4.1 Different Diel Response of the Study Systems

The peat bog was a shallow, nutrient‐poor and highly acidic water body with a large amount of decomposed organic material and had a terrestrially‐driven DOM pool. In contrast, the lake was much deeper, with clear water and its DOM comprised largely of algae‐derived compounds. These contrasting biogeochemical characteristics of our study systems had led us to hypothesise in the first place that diel light cycles would have a stronger effect on the bacterial community in the clearwater system. Specifically, we expected that significant light absorption by CDOM in the peat bog (Coble et al. 1998) would dampen diel light‐driven microbial processes, whereas in the clearwater system, photosynthetic microorganisms would more directly utilise available light. However, no diel patterns in community composition were detected in the clearwater lake during any sampling period.

We also hypothesised that diel responses in bacterioplankton communities in the clearwater system would vary with photic zone depth. Yet, the absence of diel dynamics at all depths led us to reject this hypothesis. This outcome was unexpected, particularly given the pronounced day‐night variations in illuminance observed during both sampling periods in the lake. Moreover, bacterial community composition did not significantly correlate with illuminance, as tested using the *envfit* function. Contrary to our expectations, diel light cycles influenced the bacterioplankton community structure only in the humic, low‐light‐penetration peat bog during the autumn equinox, when day and night lengths were equal. Another relevant factor to consider is that the higher CDOM concentrations in the peat bog likely caused strong attenuation of ultraviolet (UV) radiation, whereas UV penetration was deeper in the clearwater lake (Tedetti and Sempéré 2006). Since CDOM absorbs much of the biologically harmful UV radiation (Xenopoulos et al. 2000), bacterial communities in the lake may have experienced greater photostress compared to the more UV‐shielded humic waters during autumn. It is also possible that during June, sustained light exposure in both systems masked or suppressed diel patterns. Nonetheless, our findings suggest that solar irradiation may not be the primary driver of diel structuring in bacterial communities, as previously proposed (Grubisic et al. 2017; Ruiz‐González et al. 2013).

The absence of diel trends also extended to gas dynamics in the clearwater lake, where pCO_2_ and pCH_4_ fluctuations were primarily driven by stratification and mixing patterns. In the peat bog, the increase in pCH_4_ observed in September could be attributed to a precipitation event (Sanches et al. 2019). Given its small magnitude, we consider it unlikely that precipitation caused dispersal of allochthonous cells into the peat bog sufficient to influence the active fraction of the bacterial community (Wisnoski et al. 2020). Distinct diel fluctuations in pCO_2_ were observed in the peat bog during both sampling periods, with the lowest values consistently recorded at the end of daylight hours (19:15). This pattern aligns with typical metabolic diel CO_2_ dynamics, where photosynthesis reduces pCO_2_ levels during the day, while respiratory processes dominate and increase pCO_2_ at night (Rudberg et al. 2021). Chl‐*a* dynamics also showed no diel trends in June across either system, which may be explained by the disruption of periodic gene expression for chlorophyll biosynthesis under constant light conditions (Su et al. 2024). In September, under the equal light and dark periods, the clearwater lake exhibited greater variation in chl‐*a* dynamics across diel cycles. In contrast, the low concentrations of chl‐*a* in the peat bog showed no clear relationship with the light cycles or the pCO_2_ trends, suggesting that heterotrophic processes, such as organic matter respiration, could have played a more significant role in gas production.

Furthermore, fluorescence metrics showed no substantial changes in organic matter composition over diel cycles or between sampling periods in either water body. This lack of diel variation could indicate either the absence of such cycles or the limitations of our spectroscopic method in detecting subtle changes in the most labile DOM fraction. Previous studies have suggested that while DOM concentrations are often stable over time, a small, labile fraction may cycle on diel scales (Watras et al. 2015), influenced by hydrological, physical, and biological processes (Hale et al. 2024). These processes can lead to complex responses, with the importance of controlling factors shifting over time, potentially dampening or amplifying diel DOM patterns (Schwab et al. 2018). Light availability, through photodegradation, plays a critical role in shaping DOM biodegradation potential (Cory and Kling 2018), and interactions between photo‐ and biodegradation processes can influence organic compound availability to microorganisms, creating competition or cooperation among microbial pathways (Bowen et al. 2020). In June, photodegradation may have predominantly produced non‐biodegradable compounds, limiting bacterial utilisation. Alternatively, photodegradation may have generated an excess of bioavailable compounds, minimising diel effects by decoupling carbon availability from short‐term microbial responses. In September, photodegradation likely differed between systems: in the peat bog, terrestrial DOM may have been transformed into more bioavailable compounds, potentially driving diel cycling and shaping bacterial communities. In contrast, algae‐derived DOM in the lake likely exhibited weaker diel cycling, contributing to community stability. Overall, the absence of diel variation in the lake suggests that bacterial growth was not tightly coupled to the autochthonous, photosynthetically produced carbon pool (Gasol et al. 1998).

### 4.2 Diel Patterns of Peat Communities in Balanced Light Cycles

We further hypothesized that diel fluctuations in bacterial community composition would be minimal in June, when daylight prevailed for over 20 h and twilight conditions persisted during the remainder of the day. As expected, bacterial community composition remained stable over diel timescales for both the lake and the peat bog during this period. In September, however, a distinct cyclic pattern was detected in the peat bog communities, characterised by an increase in the relative abundance of certain early riser ASVs during the morning sampling time at 07:15 (time points 7, 11, 15). It was very unfortunate that the sample collected at time point 3, also sampled in the morning, had zero reads after DADA2 processing, preventing us from assessing this pattern across all diel cycles sampled. Nevertheless, the consistency observed over three consecutive days suggests a non‐random, diel‐driven pattern. This evidence supports our hypothesis for the peat bog: diel oscillations in community composition were more pronounced in September, when day and night durations were balanced, compared to the long‐day photoperiod in June. However, no such diel trends were observed in the lake during either summer or autumn, leading us to reject this hypothesis for the lake system. Intriguingly, in September, 16S ribosomal transcripts from the peat bog showed notable differences during the morning (07:15), after approximately 12 h of darkness. The night community (01:15), which had experienced only 6 h of darkness, was more similar to the solar noon (13:15) and evening (19:15) communities.

Bulk bacterioplankton responses to light are often driven by group‐specific behaviours (Ruiz‐González et al. 2013). In our study, we observed ASV‐specific diel patterns in the peat bog community during the September campaign, linked to temporal changes in their relative abundance. Some of the 12 ASVs identified as early risers (i.e., ASVs that had consistently their highest relative abundance at 07:15) could also be characterised as conditionally rare taxa (Shade et al. 2014) as, except for the 07:15 sampling times, their relative abundance was consistently less than 1% (Figure S13: ASVs 3, 6, 7, 8, 11 and 12). Furthermore, ASVs 1, 4 and 10 could only be detected at 07:15, while they were consistently absent at the other three sampling hours (13:15, 19:15, 01:15). The taxonomical assignment of the early riser ASVs indicated both phototrophic and heterotrophic lifestyles.

The only early riser undoubtedly identified as phototrophic was ASV 9, which was classified as *Cyanobium* PCC‐6307, a member of the picocyanobacterial family Cyanobiaceae. ASVs identified as *Cyanobium* PCC‐6307 have been previously reported to display diel rhythms in relative abundance, with a phase of 12 h, in coral reef seawater (Weber and Apprill 2020). It has been suggested that the circadian clock of Cyanobacteria synchronises their physiology with diel cycles in the following way: Before sunrise, Cyanobacteria initiate the transcription of genes that encode for the photosynthetic apparatus, which allows them to prepare for conditions when light is available (Bell‐Pedersen et al. 2005; Hellweger et al. 2020). This could give them a significant fitness advantage over other photosynthetic competitors (Salmela and Weinig 2019). Accordingly, one possible explanation for the observed diel shifts could be that *Cyanobium* PCC‐6307 increased its cell‐specific count of ribosomes in early morning in order to boost protein synthesis, and because of this, it was identified as an active member of the morning community. Another plausible scenario is that cell division predominantly occurs at night, as previously suggested for *Prochlorococcus* (Vaulot et al. 1995) and therefore, we detected the highest abundance of ribosomal RNA at the end of the night (i.e., in the morning). Linz et al. (2020) also observed the highest expression of photosynthetic, carbon fixation‐related, and RNA polymerase genes in the morning in a humic lake, further reinforcing the connection between diel cycles and microbial metabolic activity.

ASV9 was detected under both light regimes in both water bodies, but a diel pattern in its relative abundance was observed only in the peat bog during September. We speculate that in June, when nights are short and not completely dark, maintaining relatively constant protein synthesis throughout the day may provide a selective advantage, even for photosynthetic organisms. The absence of diel ribosomal RNA dynamics in *Cyanobium* PCC‐6307 strains during the long‐day photoperiod could be attributed to either external factors, such as the lack of strong environmental regulatory cues like prolonged dark periods, or internal factors, such as the dominance of populations without internal circadian clocks. Interestingly, no diel trend for ASV9 was detected in the lake during September, despite experiencing diel light patterns similar to those of the peat bog. This suggests that the observed diel fluctuation of ASV9 in the autumn peat bog may result from specific interactions involving *Cyanobium* PCC‐6307 and other biotic or abiotic factors unique to the bog environment. Supporting this, we observed a significant positive correlation between the relative abundance of *Cyanobium* PCC‐6307 with the pCO_2_ and pCH_4_ in the peat bog in September (Figure 5B). Under the oxic conditions of the peat bog, it is possible that the higher morning abundance of *Cyanobium* PCC‐6307 is linked to their direct involvement in methane production (Fazi et al. 2021). There is emerging evidence that cyanobacteria can convert fixed inorganic carbon into methane under both light and dark periods (Bižić et al. 2020), but they can also produce this gas by demethylation of methylphosphonates (Yao et al. 2016). The latter process could be supported by the availability of labile carbon sources (Peoples et al. 2023), indicated by the significant negative correlation of the relative abundance of *Cyanobium* PCC‐6307 with the less labile terrestrially driven DOM depicted in peak C PARAFAC component. Overall, the observed diel fluctuation of *Cyanobium* PCC‐6307 seemed to have been strongly linked to its metabolic cycles, including its day‐night photosynthetic activity, which in turn could have contributed to the simultaneously measured diel pCO_2_ dynamics.

In addition to the diel periodicity of photoautotrophic microorganisms, complex interactions within the aquatic food web may influence community‐level diel dynamics. Heterotrophic bacteria, for instance, can respond to pulses of organic matter released from primary producers, viral lysis, or grazing (Morimoto et al. 2020; Ruiz‐González et al. 2012; Sadro et al. 2011). Bacterial peat bog communities in September sampling significantly correlated positively with HIX and pCH_4_, and negatively with the T and B peaks, which are indicative of more bioavailable organic material of microbial or phytoplankton origin (Fellman et al. 2010). Simultaneously, the early riser ASVs correlated negatively with dissolved oxygen, HIX and the humic‐like C and M peaks, and positively with pCO_2_ and pCH_4_. Consequently, it is likely that diel changes in the metabolism of the humic‐rich organic material could be the underlying factor of the diel fluctuations of at least some of the heterotrophic early risers.

For instance, six early riser ASVs (ASV3‐8) were classified as Bacteroidia, a class broadly recognised for its ability to degrade complex biopolymers (Kirchman 2002) and typically profit from terrestrial or autochthonous dissolved organic carbon (Eiler and Bertilsson 2007; Zeder et al. 2009). Using CARD–FISH, Ruiz‐González et al. (2012) detected diel variations in the abundance of Bacteroidetes, which showed significantly higher contributions during the day in one of the observed diel cycles. Metatranscriptomic studies have also provided some evidence of diel expression cycles in non‐photosynthetic members of the phylum Bacteroidota living in microbial mats alongside Cyanobacteria (Hörnlein et al. 2018). In this context, the early riser Bacteroidia ASVs could simply respond to the diel influx of fixed carbon and oxygen resulting from the photosynthesis of other members of the peat bog community, like *Cyanobium* PCC‐6307. This way, the diel dynamics of photoautotrophs may have had cascading effects on the heterotrophic fraction of the community (Linz et al. 2020; Morimoto et al. 2020) by providing a diel influx of fixed carbon and oxygen.

The abundance of ASV11, belonging to the SAR11 clade, also correlated positively with pCO_2_ and pCH_4_, but not with the humic substances related to the PARAFAC peaks. SAR11 has been shown to exhibit diel variations in single‐cell activity despite relatively stable abundances throughout the diel cycle (Ruiz‐González et al. 2012). Moreover, a strain of SAR11 has been found to produce methane through the decomposition of methylphosphonic acid under phosphate starvation (Carini et al. 2014) and we speculate that this could also happen in the phosphorus‐limited peat bog system. Concurrently, the abundance of ASV2, an hgcI clade member, exhibited strong positive correlations with precipitation, which could reflect that these Actinobacteria benefited from pulses of terrestrial organic matter in the peat bog (Ghai et al. 2014). As Ottesen et al. (2014) suggested for marine environments, synchronous biogeochemical processes on diel timescales among different heterotrophic bacteria could regulate the matter and energy transformation in freshwater ecosystems. The observed pCO_2_ cycles suggest that synchronised heterotrophic activity, driven by early risers, likely dominated diel community dynamics in the peat bog during autumn.

Lastly, an interesting result was that the relative abundance of the 12 early riser ASVs did not exhibit significant correlations with water temperature. Circadian rhythms follow temperature compensation; namely, a constant rhythm can be maintained within a certain range of temperatures, in contrast to a typical biochemical reaction (Bodenstein et al. 2012). This could explain the absence of an effect in community composition related to temperature variability if any of these ASVs possess a circadian clock mechanism. The fact that light and temperature variability alone could not explain the observed diel patterns suggests that more complex biotic interactions may occur at the community‐level. Rather than being uniformly stimulating or detrimental, sunlight can have a range of effects on bacterial activity, and this complexity renders it difficult to predict microbial community responses based solely on taxonomic or physiological traits (Ruiz‐González et al. 2013). Diel community shifts in the peat bog were likely influenced by a combination of phototrophic processes responding to light cycles and heterotrophic processes potentially driven by the availability of organic substrates. This could account for why the diel response differed between the peat bog and the lake under a light regime with equal day–night length. Last, the possibility exists that diel metabolic responses of certain taxa could not be captured by only sequencing the 16S rRNA (Blazewicz et al. 2013). To deepen our understanding of metabolic, biogeochemical and ecological processes driving diel shifts in microbial ecosystems, we advocate for future diel studies in natural bacterial communities using metatranscriptomic and metabolomic approaches under different light regimes and at different latitudes.

## 5 Conclusions

Our study, utilising amplicon sequencing of 16S ribosomal RNA transcripts, revealed that during the boreal summer, close to the summer solstice, bacterioplankton communities in both a clearwater oligotrophic lake and a humic bog did not exhibit diel changes under conditions of prolonged daylight. Similarly, in the lake, community structure remained stable throughout the autumn equinox when the day–night length was balanced. However, in the peat bog during the same period, cyclic fluctuations were observed, with specific photo‐ and heterotrophic ASVs showing a characteristic increase in relative abundance in the morning. These short‐term bacterioplankton population dynamics provide evidence of ecological niches shaped by both the overall day–night cycle and the specific time of day. While the light regime in the boreal region is expected to remain relatively constant, the aquatic boreal microbiome may respond differently to new abiotic diel fluctuations, such as increased temperatures. Given the crucial role of boreal freshwater systems in carbon cycling and the ongoing impacts of climate change in the region, it is essential to continue investigating how bacterial taxa respond to diel patterns, whether common diel trends exist across waterbodies of similar trophic status, and how community interactions may drive these diel fluctuations for specific taxa. Despite the ecological importance of these systems, diel microbial studies in the boreal region remain scarce, highlighting the need for further research in these environments.

## Author Contributions

**Sofia Papadopoulou:** validation, formal analysis, investigation, data curation, writing – original draft, visualization. **Annika Linkhorst:** investigation, methodology, validation, writing – review and editing. **John Paul Balmonte:** methodology, validation, investigation, writing – review and editing. **Bianka Csitári:** investigation, writing – review and editing. **Tamás Felföldi:** investigation, writing – review and editing. **Zsuzsanna Márton:** investigation, writing – review and editing. **Maliheh Mershad:** investigation, writing – review and editing. **Attila Szabó:** investigation, writing – review and editing. **Anders Torstensson:** investigation, writing – review and editing. **Stefan Bertilsson:** resources, writing – review and editing. **Anna J. Székely:** conceptualization, methodology, validation, formal analysis, investigation, data curation, writing – review and editing, visualization, supervision, project administration, funding acquisition.

## Conflicts of Interest

The authors declare no conflicts of interest.

## Supporting information


**Data S1.** Supporting Information.

## Data Availability

The raw sequencing data for this study have been deposited in the European Nucleotide Archive (ENA) at EMBL‐EBI under accession number PRJEB62428.
