# Supplementary material for: Diel Bacterioplankton Community Dynamics Under Contrasting Light Regimes
Source: Environ Microbiol Rep. 2025 May 8;17(3):e70099. doi: 10.1111/1758-2229.70099 (PMC12061850; doi:10.1111/1758-2229.70099)
Supplement: Supplementary file 1 — Data S1. Supporting Information. [file EMI4-17-e70099-s001.docx]

**Supplementary data and methods for**:

**Diel bacterioplankton community dynamics under contrasting light regimes**

Sofia Papadopoulou ^a *^, Annika Linkhorst ^a b^, John Paul Balmonte ^a c^, Bianka Csitári ^a d e^, Tamás Felföldi ^d f^, Zsuzsanna Márton ^d f^, Maliheh Mershad ^a g^, Attila Szabó ^d f g^, Anders Torstensson ^a h^, Stefan Bertilsson ^a g^, Anna J. Székely ^a g^

^a^ Department of Ecology and Genetics/Limnology, Uppsala University, Uppsala, Sweden

^b^ Department of Environmental Radioactivity and Monitoring, Federal Institute of Hydrology (BfG), Koblenz, Germany

^c^ Lehigh Oceans Research Center, Lehigh University, Bethlehem, PA, USA

^d^ Department of Microbiology, ELTE Eötvös Loránd University, Budapest, Hungary

^e^ Department of Microbiology, Tumor and Cell Biology, Karolinska Institute, Stockholm, Sweden

^f^ Institute of Aquatic Ecology, HUN-REN Centre for Ecological Research, Budapest, Hungary

^g^ Department of Aquatic Sciences and Assessment, Swedish University of Agricultural Sciences, Uppsala, Sweden

^h^ Swedish Meteorological and Hydrological Institute, Community Planning Services - Oceanography, Västra Frölunda, Sweden

* Corresponding author: [sofia.papadopoulou@ebc.uu.se](mailto:sofia.papadopoulou@ebc.uu.se)

*Study systems*


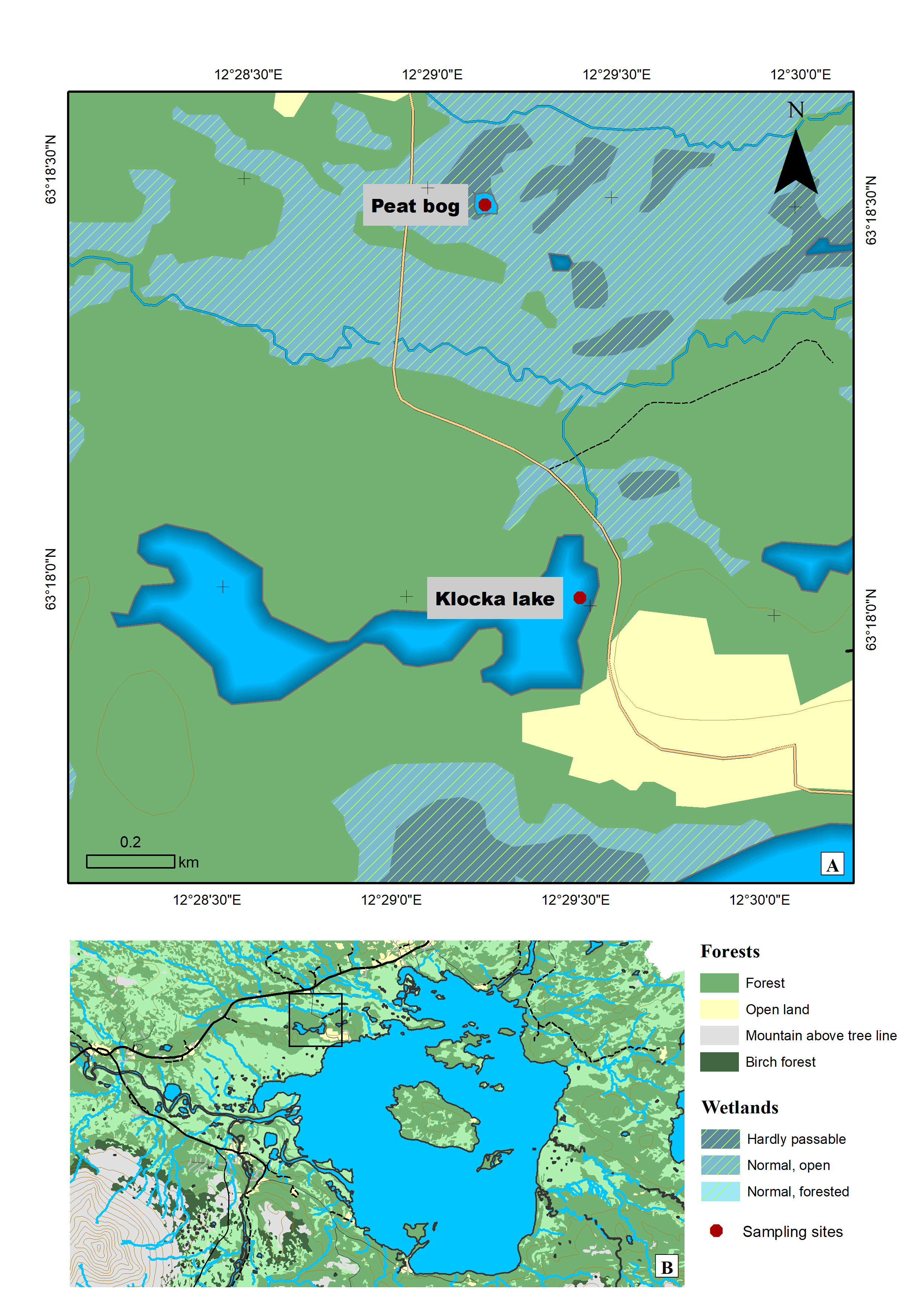

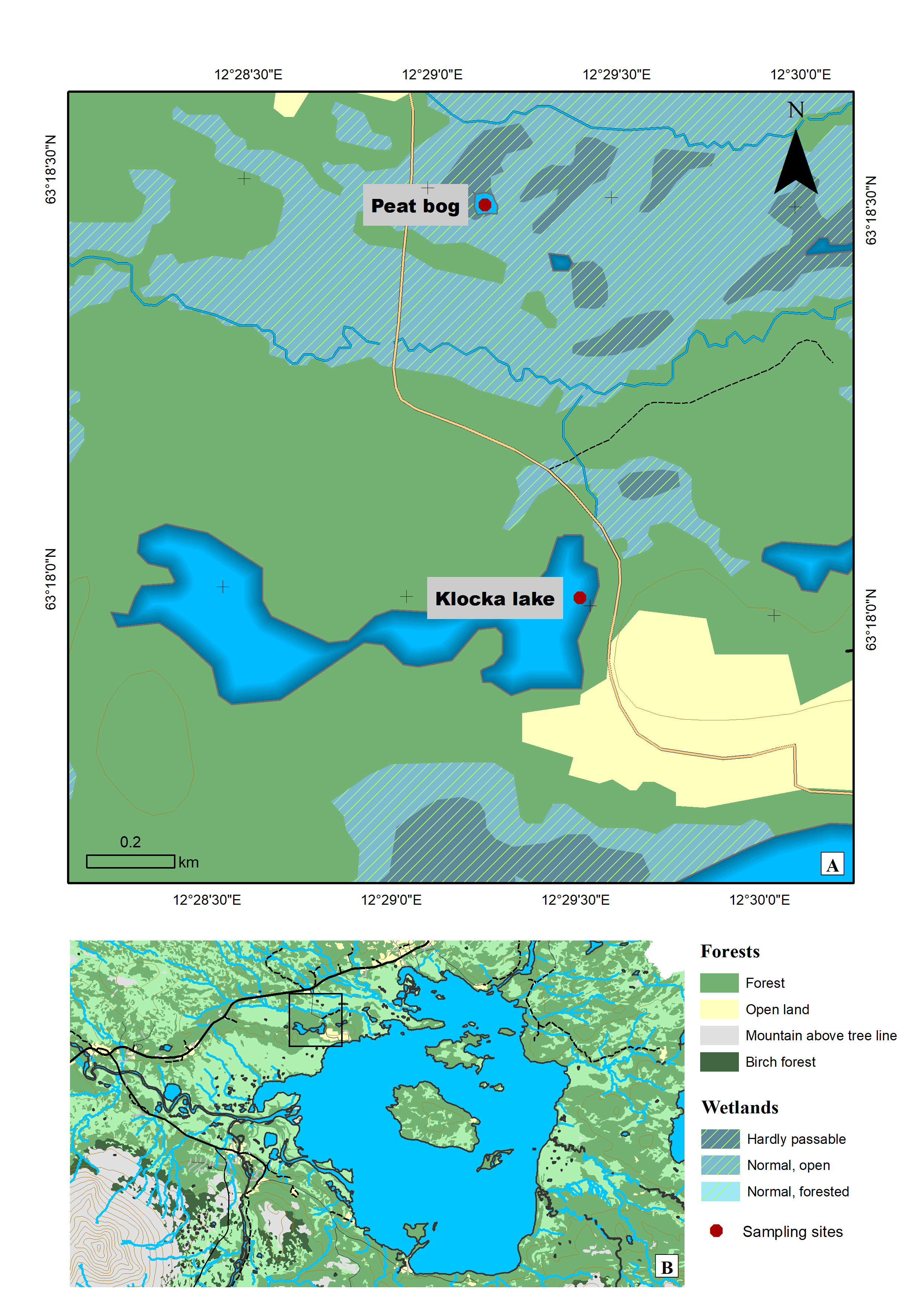

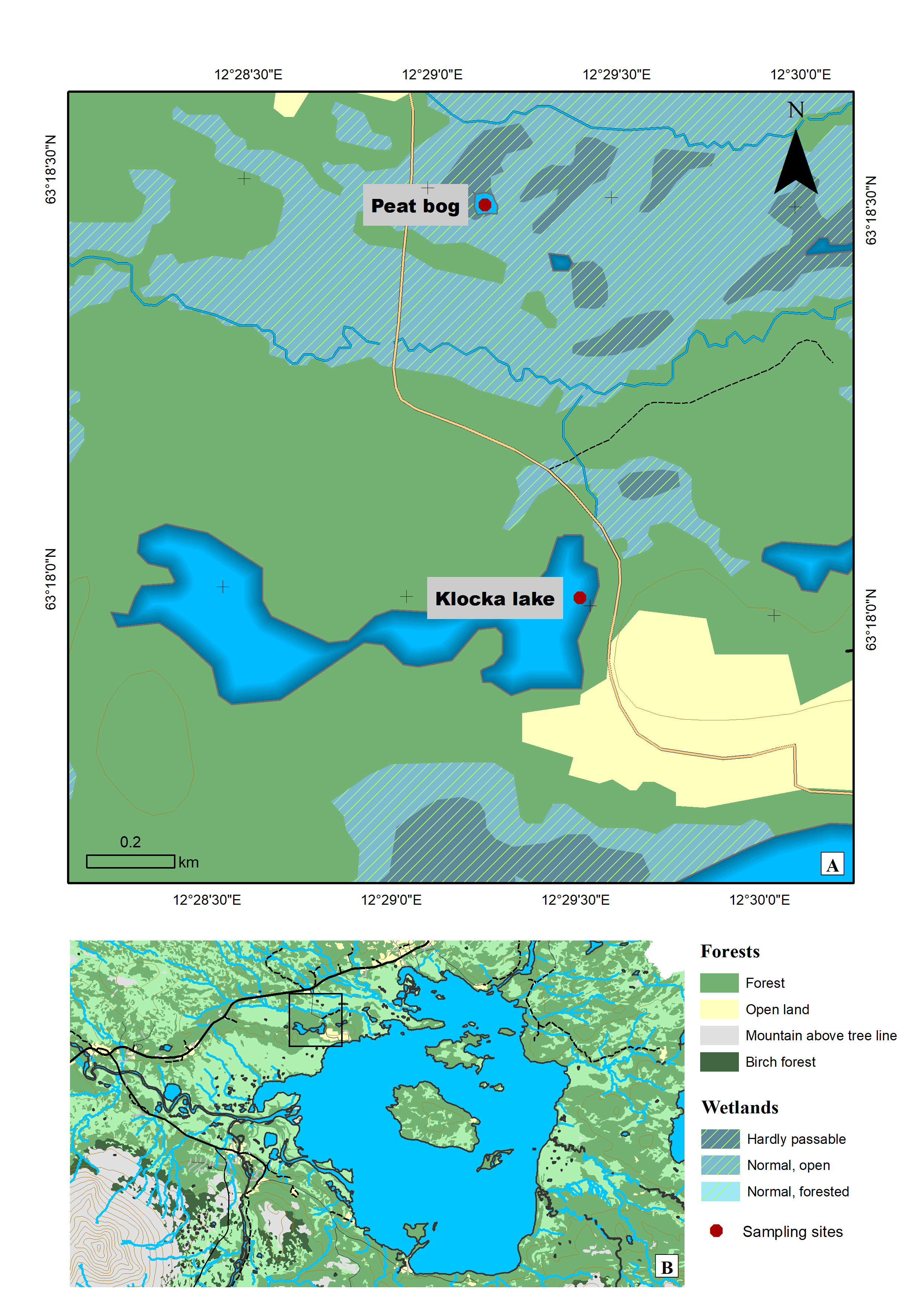

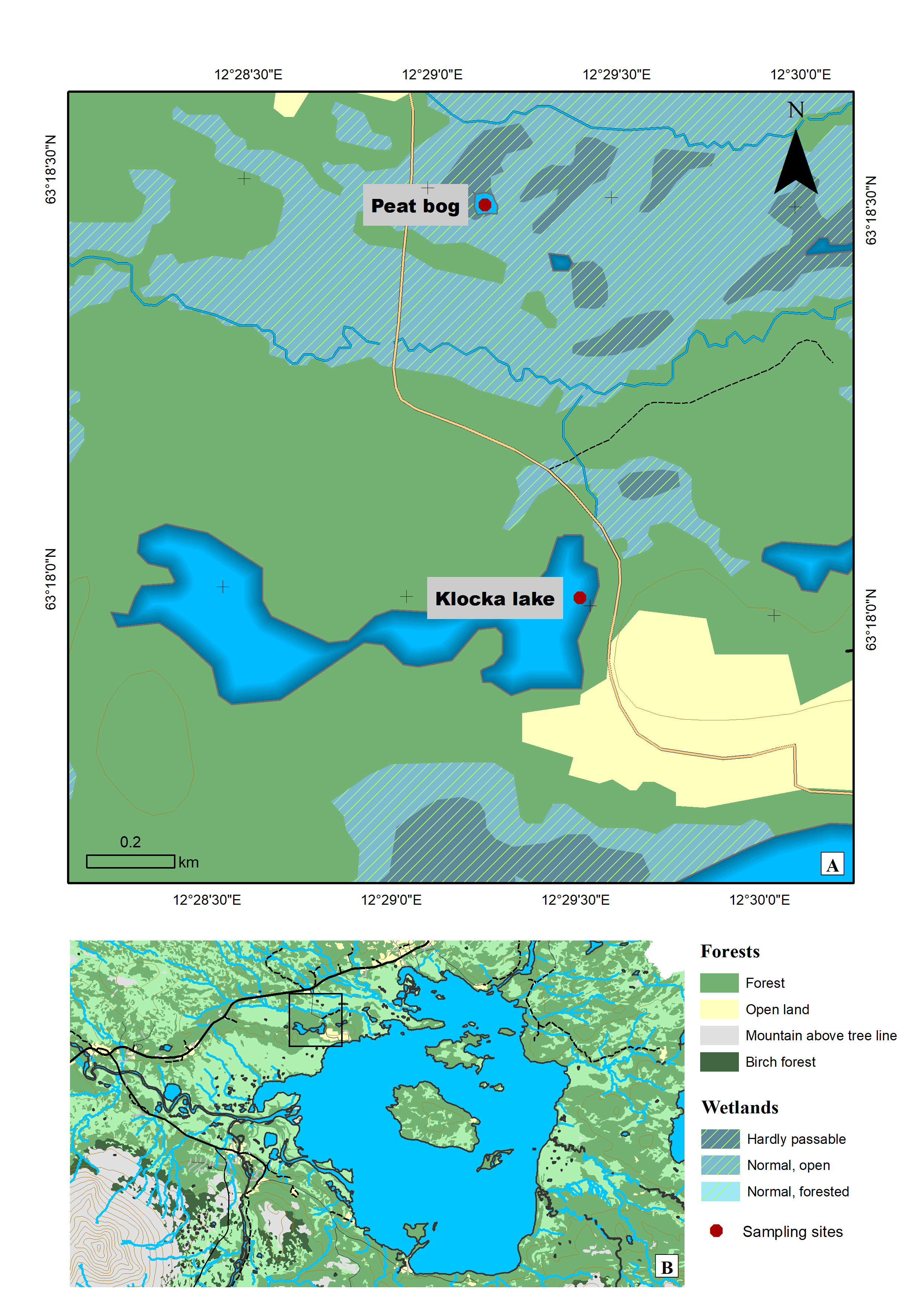


**Figure S1.** A. Location of the two study systems, lake Klocka (Klocktjärnen) and the peat bog. B. Relative location of the study systems to the Ånnsjön lake of Jämtland county, Sweden. The map was created with ArcMap GIS software (version 10.8) and the data were retrieved from the service Geodata Extraction Tool, hosted by the Swedish University of Agricultural Sciences.

*Study systems*


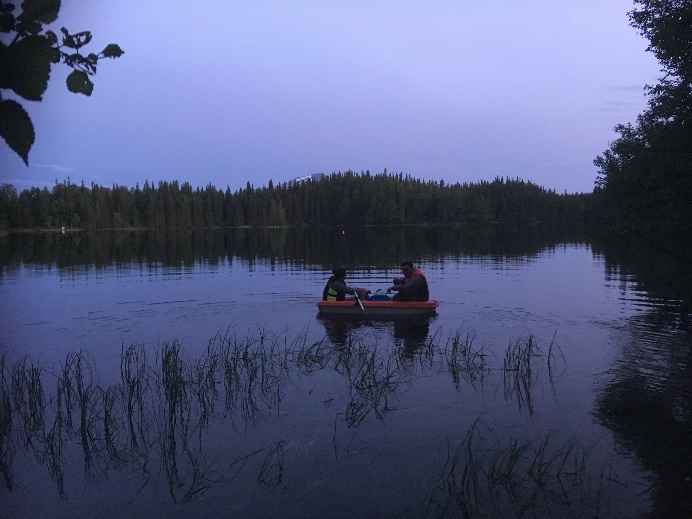


**Figure S2.** Twilight conditions in lake Klocka on 25/06/2019 at 00:59, captured with an Apple iPhone SE, Rear Camera (29 mm, ƒ2.2). Image settings: ISO 200, 0 EV, 1/17 s.

*Sun path diagram*


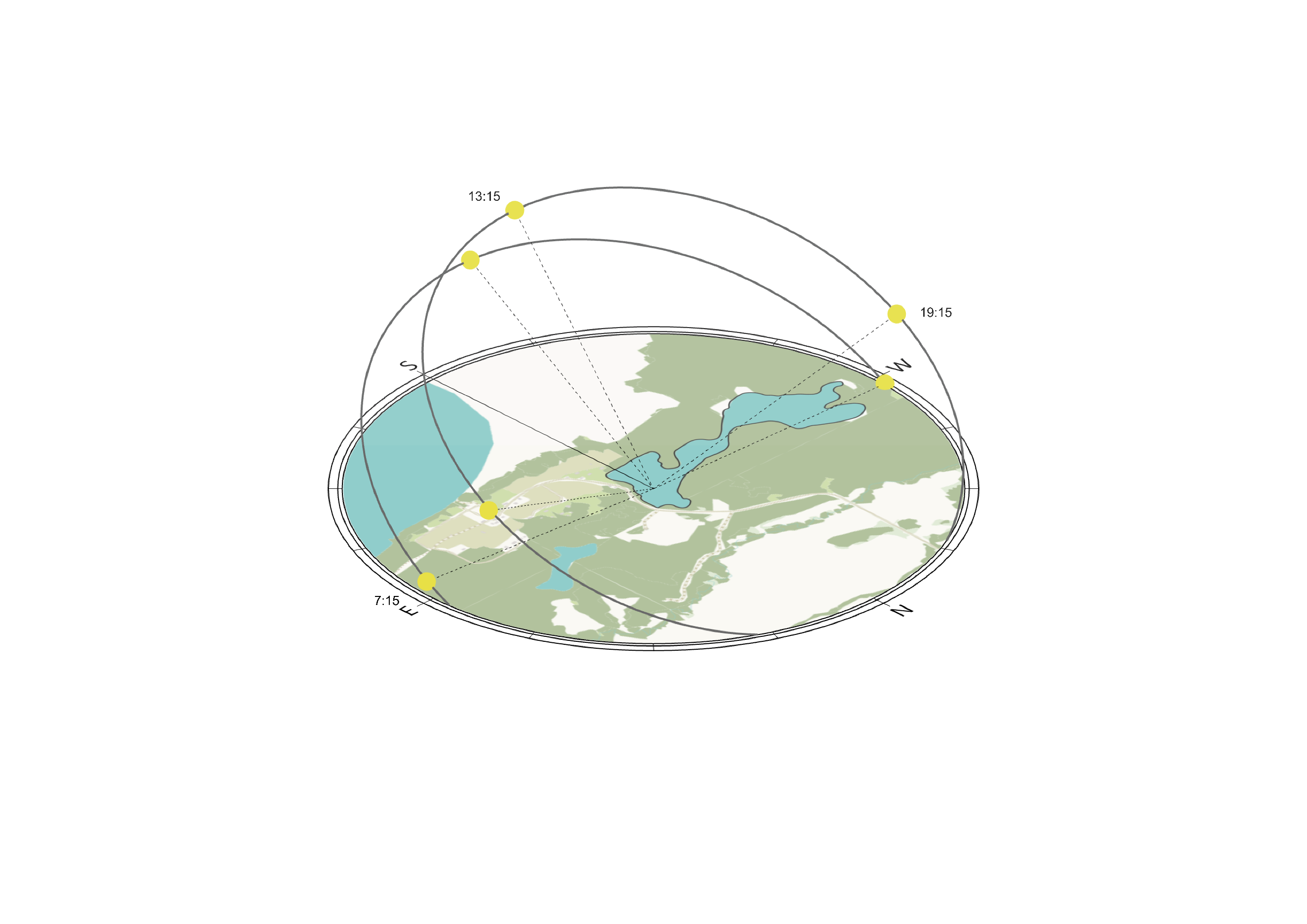


**Figure S3.** Two sun path diagrams that show the position of the Sun above the study systems at the different sampling hours during the sampling campaigns in June (upper arc) and September (lower arc). Three out of four sampling points are illustrated (07:15, 13:15, 19:15) since at 01:15 the Sun was behind the horizon. The local solar noon, when the Sun reached its highest position in the sky, was at 13:15.

*Lake stratification*


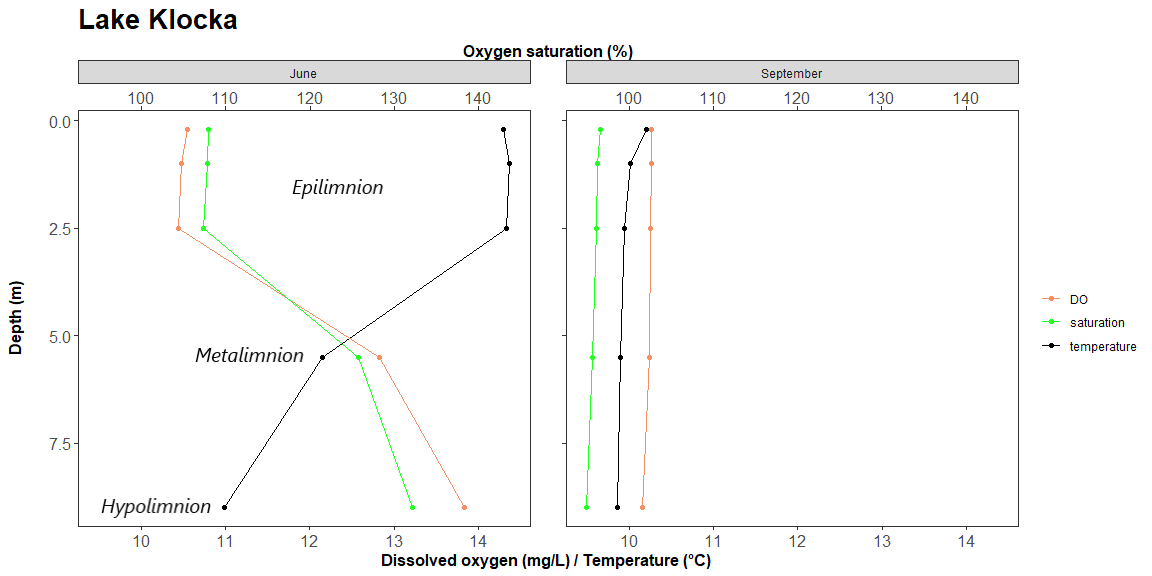


**Figure S4.** Illustration of mean values of water temperature, dissolved oxygen (DO) concentration and its saturation in the lake during the two sampling periods. The lake was stratified in June and mixed in September.

**Table S1.** Mean values of physicochemical measurements (temperature, dissolved oxygen concentration and its saturation, electrical conductivity and pH in the peat bog and lake Klocka during the two sampling periods. The variability of the mean values is represented by the standard deviation (mean ± SD). The values are presented per sampling depth when the lake was stratified in June and averaged for the whole water column in the mixed lake in September.

| Site | Temperature (^o^C) | DO (mg/L) | Saturation (%) | Conductivity (μS/cm) | pH |
| --- | --- | --- | --- | --- | --- |
| **June** | | | | | |
| Peat bog | 15.14±2.15 | 9.75±0.21 | 102.21±4.72 | 17.1±1.4 | 4.41±0.05 |
| Lake (0.2 m) | 14.29±0.62 | 10.55±0.16 | 107.91±1.98 | 176.17±2.01 | 8.22±0.07 |
| Lake  (1 m) | 14.37±0.4 | 10.48±0.13 | 107.86±1.74 | 175.7±1.69 | 8.02±0.12 |
| Lake  (2.5 m) | 14.33±0.26 | 10.44±0.12 | 107.44±1.9 | 175.51±1.43 | 8.05±0.1 |
| Lake  (5.5 m) | 12.15±0.33 | 12.82±0.3 | 125.8±3.46 | 171.79±1.68 | 8.07±0.13 |
| Lake  (9m) | 10.99±0.21 | 13.83±0.34 | 132.13±3.37 | 168.34±1.84 | 8.2±0.16 |
| **September** | | | | | |
| Peat bog | 8.22±1.02 | 10.75±0.33 | 96.85±1.3 | 25±0.83 | 4.37±0.11 |
| Lake | 9.99±0.25 | 10.24±0.17 | 95.94±1.21 | 170.5±3.6 | 8.44±0.14 |

*Nutrient measurements*

**Table S2.** Nutrient measurements for the peat bog and lake Klocka during the September sampling period. “TP” stands for total phosphorus, “TN” for total nitrogen, “N:P” is the ratio of TP to TN and “TOC” stands for total organic carbon measurements.

| **Site** | **Depth (m)** | **TP (μg/L)** | **TN (μg/L)** | **N:P** | **TOC (mg/L)** |
| --- | --- | --- | --- | --- | --- |
| Peat bog | 0.2 | 2.36 | 275.1 | 116.46 | 17.4 |
| Lake | 0.2 | 2.1 | 117 | 55.72 | 2.13 |
| Lake | 1 | 2.3 | 132.8 | 57.82 | 2.21 |
| Lake | 2.5 | 2.72 | 123.1 | 45.21 | 2.3 |
| Lake | 5.5 | 2.49 | 129.1 | 51.78 | 2.17 |
| Lake | 9 | 2.3 | 133.5 | 58.13 | 2.17 |

*High-frequency measurements of temperature and illuminance*


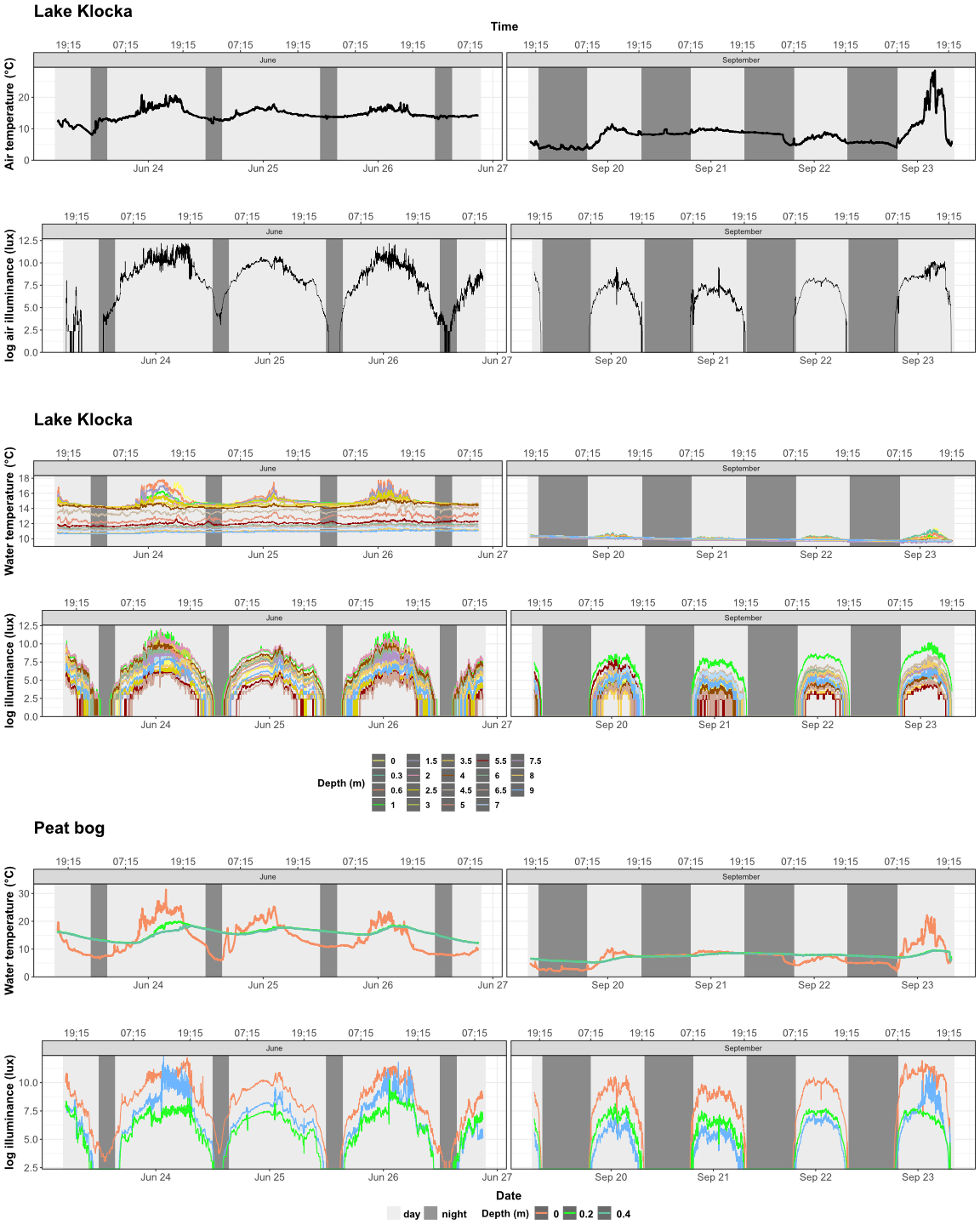


**Figure S5.** High-frequency data, measured with HOBO loggers, for the temperature and illuminance in the air above the surface and the water of the study systems during the two sampling periods. Illuminance data are transformed using the natural logarithm. “Jun” stands for June and “Sep” for September.

*Weather conditions*


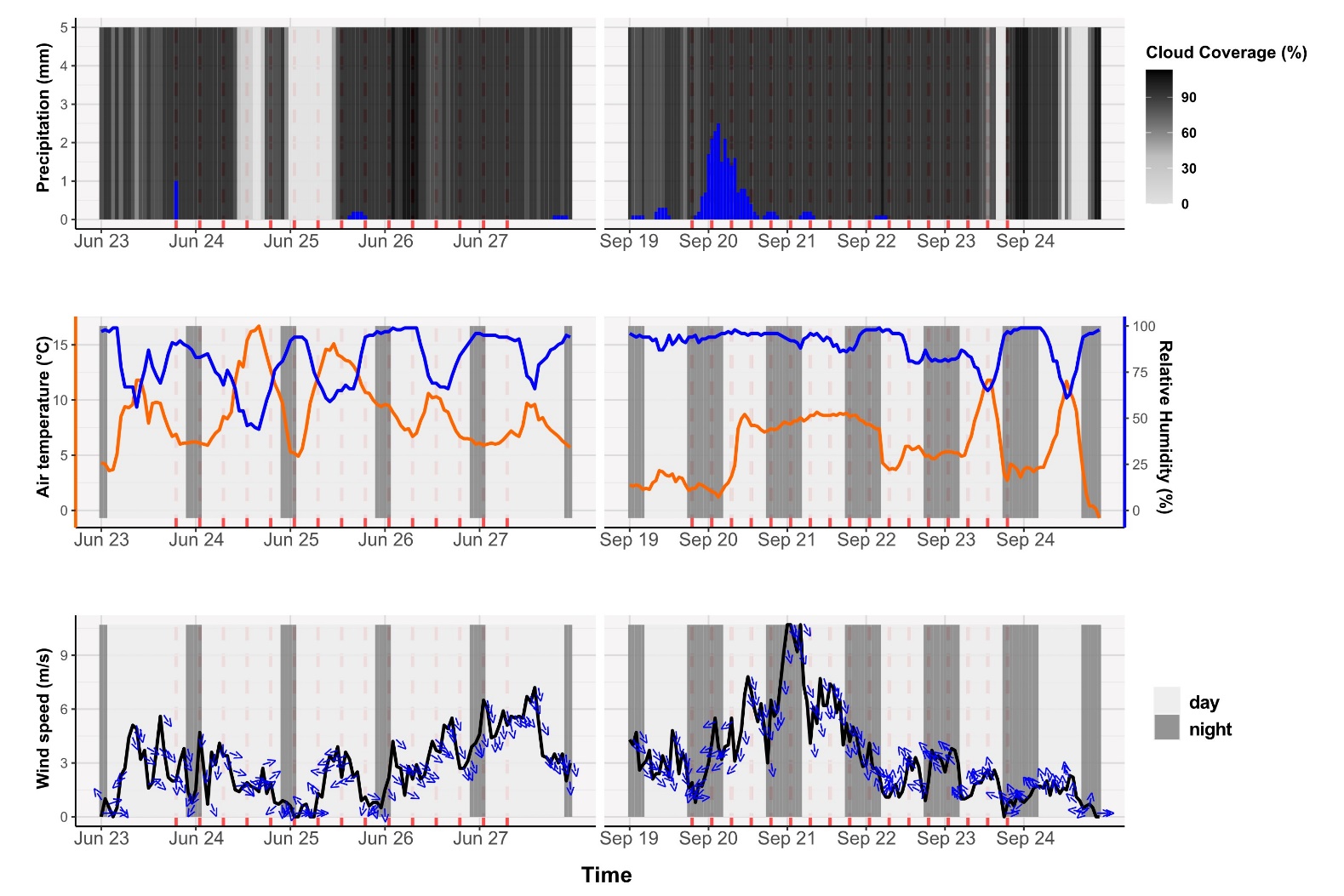


**Figure S6.** Weather conditions during the two sampling campaigns. Upper plot: Precipitation (blue) and cloud coverage (grey scale). Middle plot: Air temperature (orange) and relative humidity (blue). Lower plot: Wind speed (black) and wind direction (blue arrows). The horizontal axis shows the dates per sampling period and the red vertical dotted lines illustrate the exact time points sampled. “Jun” stands for June and “Sep” for September.

*Chlorophyll a measurements*


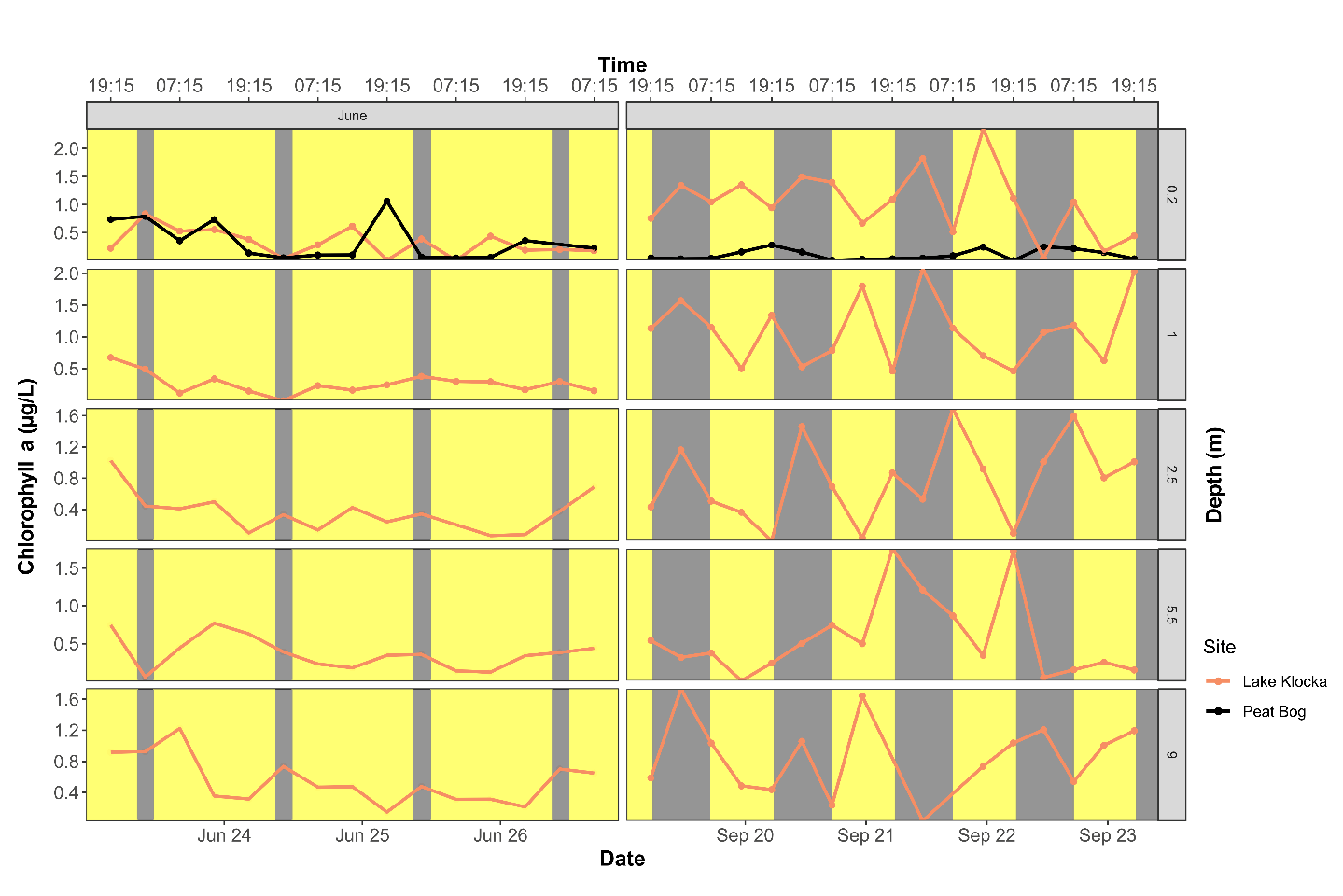


**Figure S7.** Chlorophyll *a* concentration over diel cycles in the lake and the peat bog in June and September sampling periods.

*Fluorescence components and spectral indices*


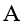

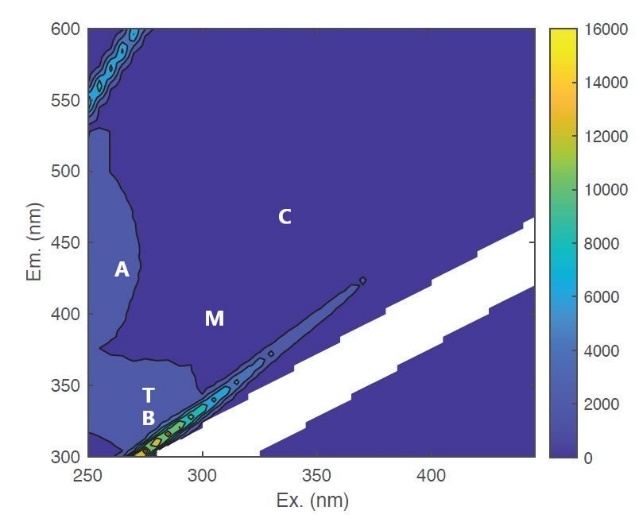

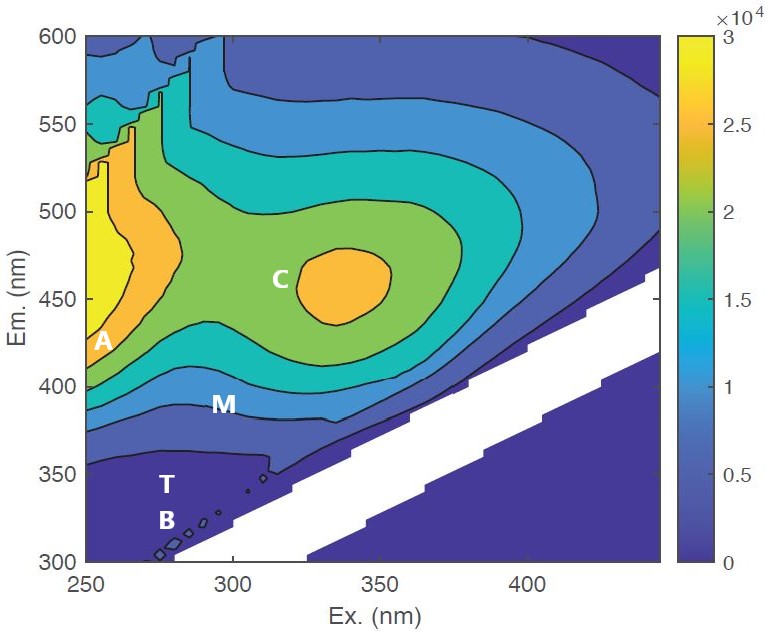


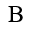


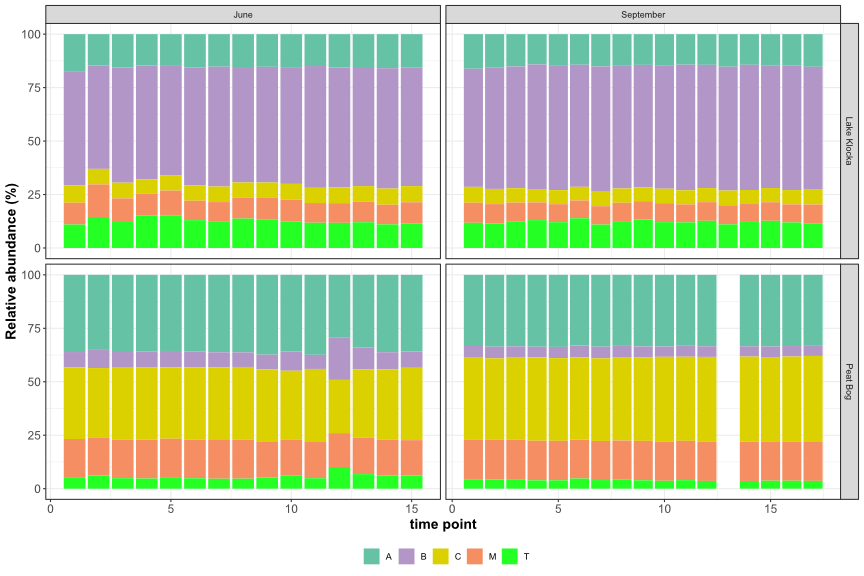


**Figure S8.** A. Example of two Excitation Emission Matrices (EEMs) showing the position of the five identified peaks (i.e., A, B, C, M, T) in optical space. Left: Lake (June, surface water, time point 7). Right: peat bog (June, time point 10). The white area in the lower right corner of each EEM is where excess scatter is removed. Fluorescence intensities are in Raman units. B. Relative abundance of peaks associated with the five fluorescence components identified in the two study systems. Relative abundance is presented over time points for each sampling period.

*Data retention through DADA2 pipeline*

A total of 8,947,622 reads for the 192 samples were initially obtained and introduced to DADA2 pipeline. Excepting three samples with less than 7,000 reads, read counts per sample averaged at 47,091 reads, with a maximum of 77,446 and a minimum of 21,718. Forward reads were truncated at position 280 (the last 20 nucleotides were trimmed), while reverse reads at position 220 (the last 80 nucleotides) as their quality was poorer. Only 56.39% of the initial input sequences were merged and 40.85% were identified as non-chimeric, resulting in 3,655,133 reads that were grouped into 87,485 ASVs. Sequences were rarefied to 7,468 reads per sample and 52,285 ASVs remained for the analysis of beta diversity. Out of the 192 samples processed with DADA2, three samples (peat bog, September, time point 3; lake, June, depth 1 m, time point 10; lake, June, depth 9 m, time point 13) had between 0 and 7,000 reads and were excluded from further analysis.

Rarefaction curves constructed per site and sampling period (Figure S9) reached a plateau for the majority of the summer lake samples and autumnal peat bog samples, indicating that sequencing depth was adequate for most of these communities.

*Rarefaction curves*


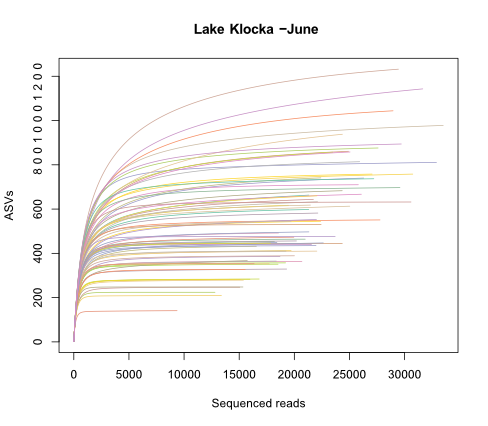

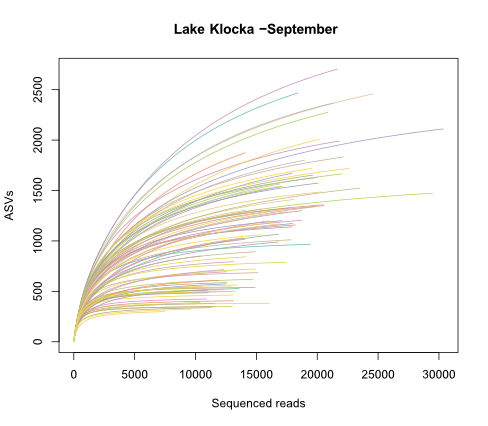


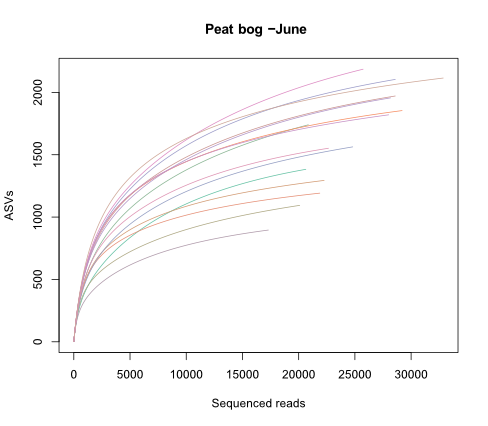

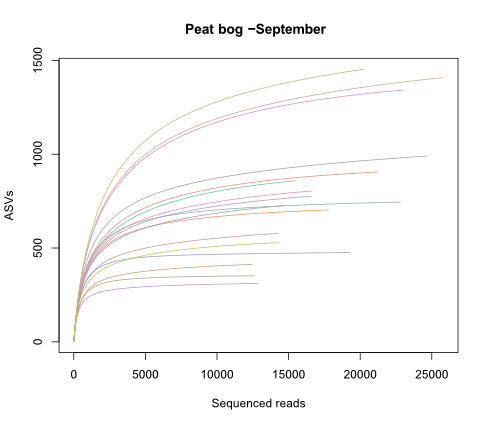


**Figure S9.** Rarefaction curves per site and sampling period.

*Shannon index*


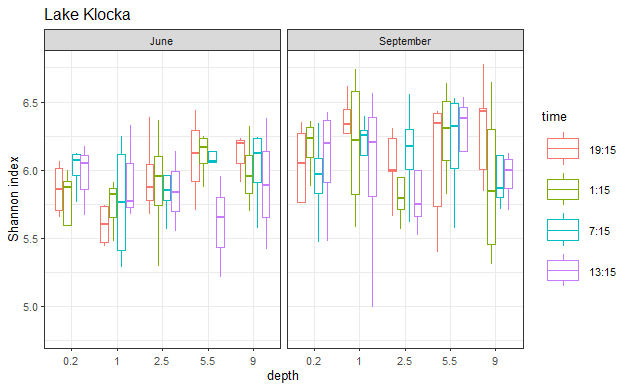


**Figure S10.** Boxplots for the Shannon index of each sampling depth in lake Klocka per month. Different colors represent the sampling time. The whiskers in the boxplots show the two opposite ends of the data that represent the spread of different samples. The line in the boxplots represents the median of the spread of the data. For the construction of this figure, ASV matrices that corresponded to the lake site were rarefied without replacement to 7,468 reads.


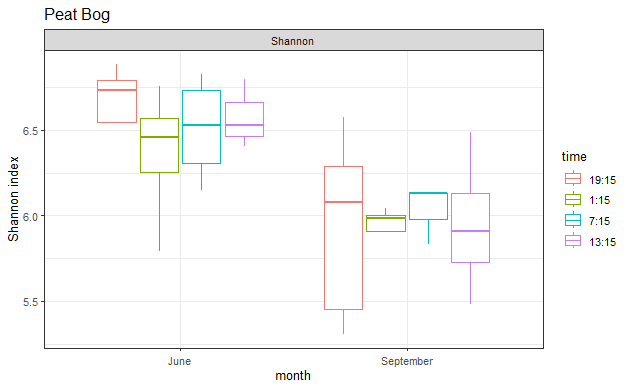


**Figure S11.** Shannon index per month for the peat bog communities. Different colors represent the sampling time. The whiskers in the boxplots show the two opposite ends of the data that represent the spread of different samples. The line in the boxplots represents the median of the spread of the data. For the construction of this figure, ASV matrices that corresponded to the peat bog site were rarefied without replacement to 12,474 reads.

**Figure S12 (next page).** Variation of bacterial community composition in the peat bog and the different sampling depths of the lake across time points in June and September sampling events. Bars represent individual samples and each color illustrates a different class of Bacteria. Taxa with relative abundances less than 1% in a sample are grouped together.

*Relative abundance of bacterial classes*


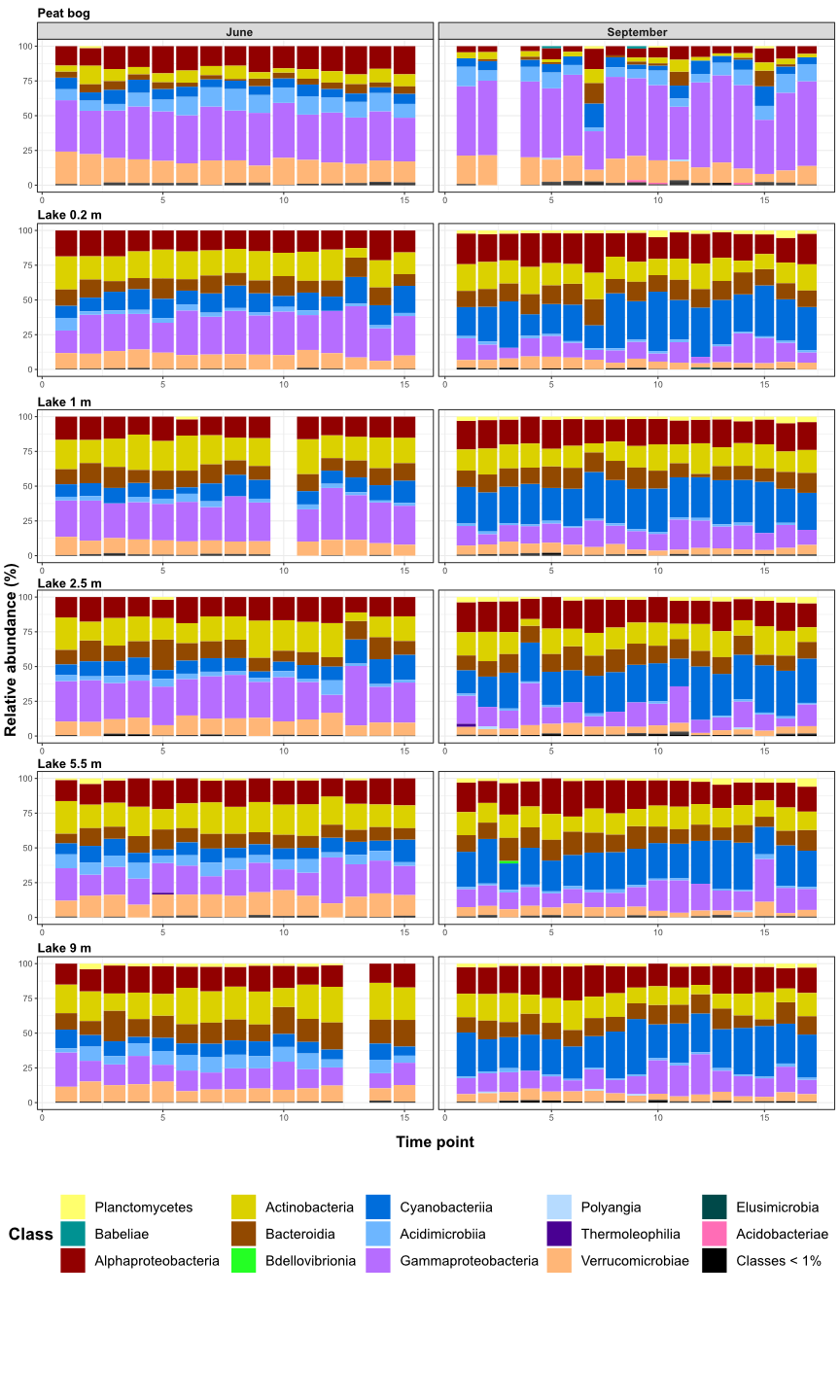


*Relative abundance of bacterial families in the peat bog*

*
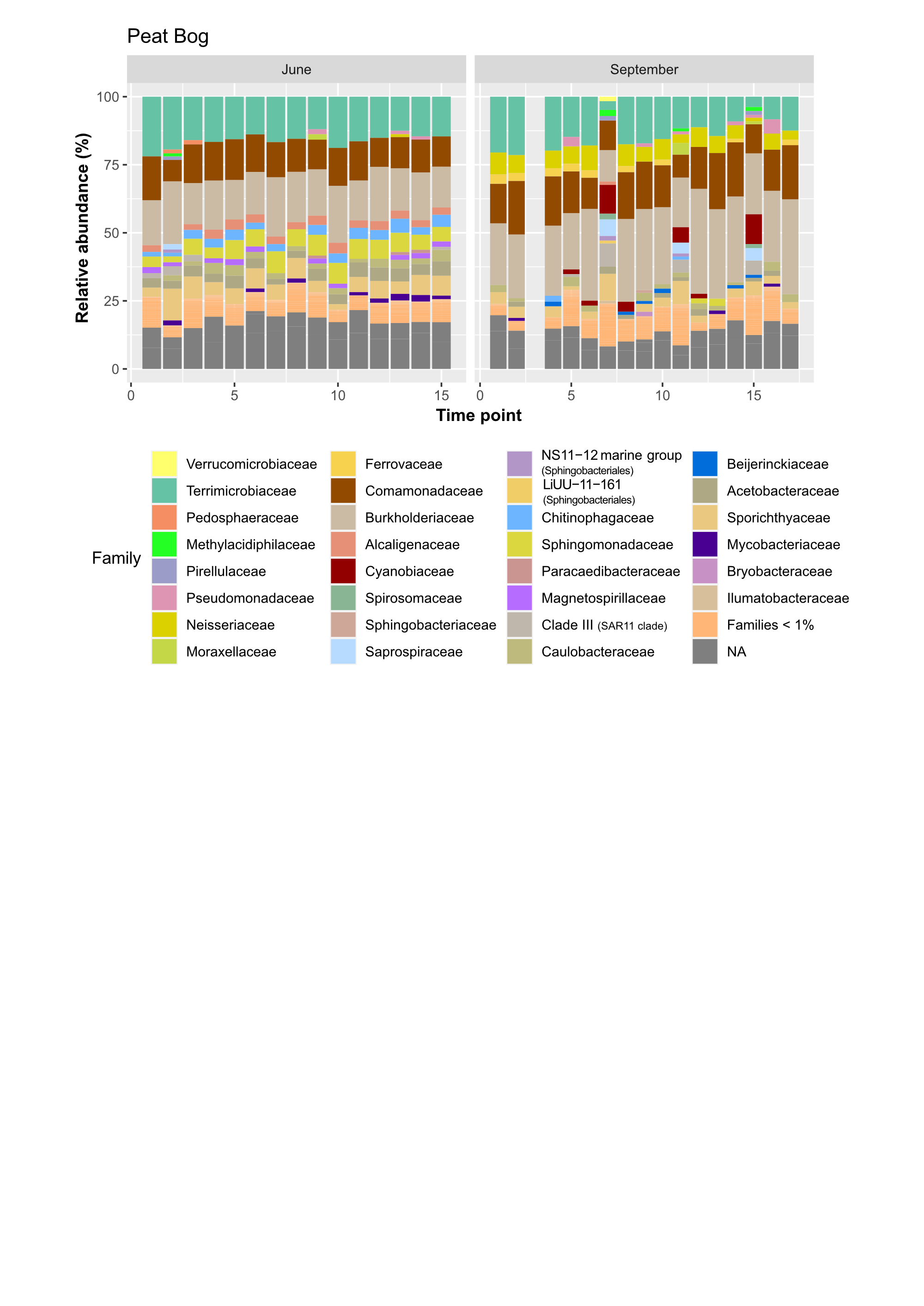
*

**Figure S13.** Relative abundance of bacterial families in the peat bog, both sampling periods. Bars represent individual samples and each color illustrates a different family of Bacteria. Taxa with relative abundances less than 1% in a sample are grouped together. ASVs with unassigned taxonomy on the family rank (bootstrap less than 50) are labelled as “NA”.

*Relative abundance of peat bog early riser ASVs*

**Figure S14 (next page).** Radar charts showing the relative abundance (%) of the twelve early riser ASVs from Table 4 over time, during the sampling campaigns in June and September. The time points are symbolized with a capital T and their respective number. Time progress is illustrated clockwise. ASV10 was not detected in June and thus its abundance was zero at all time points. The sample at time point 3 in September had zero reads and this is why T3 is missing from the autumn plots.

*
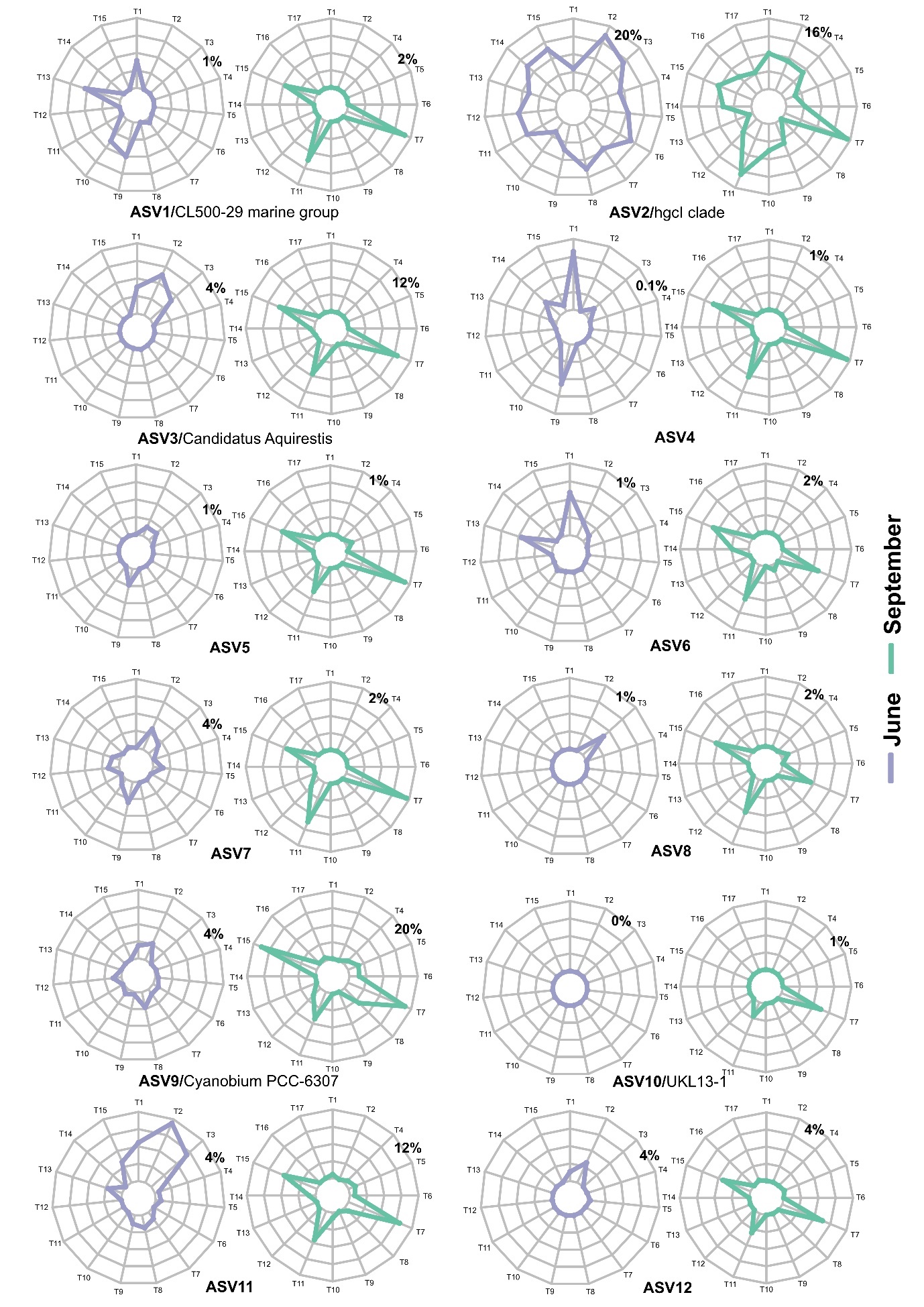
*
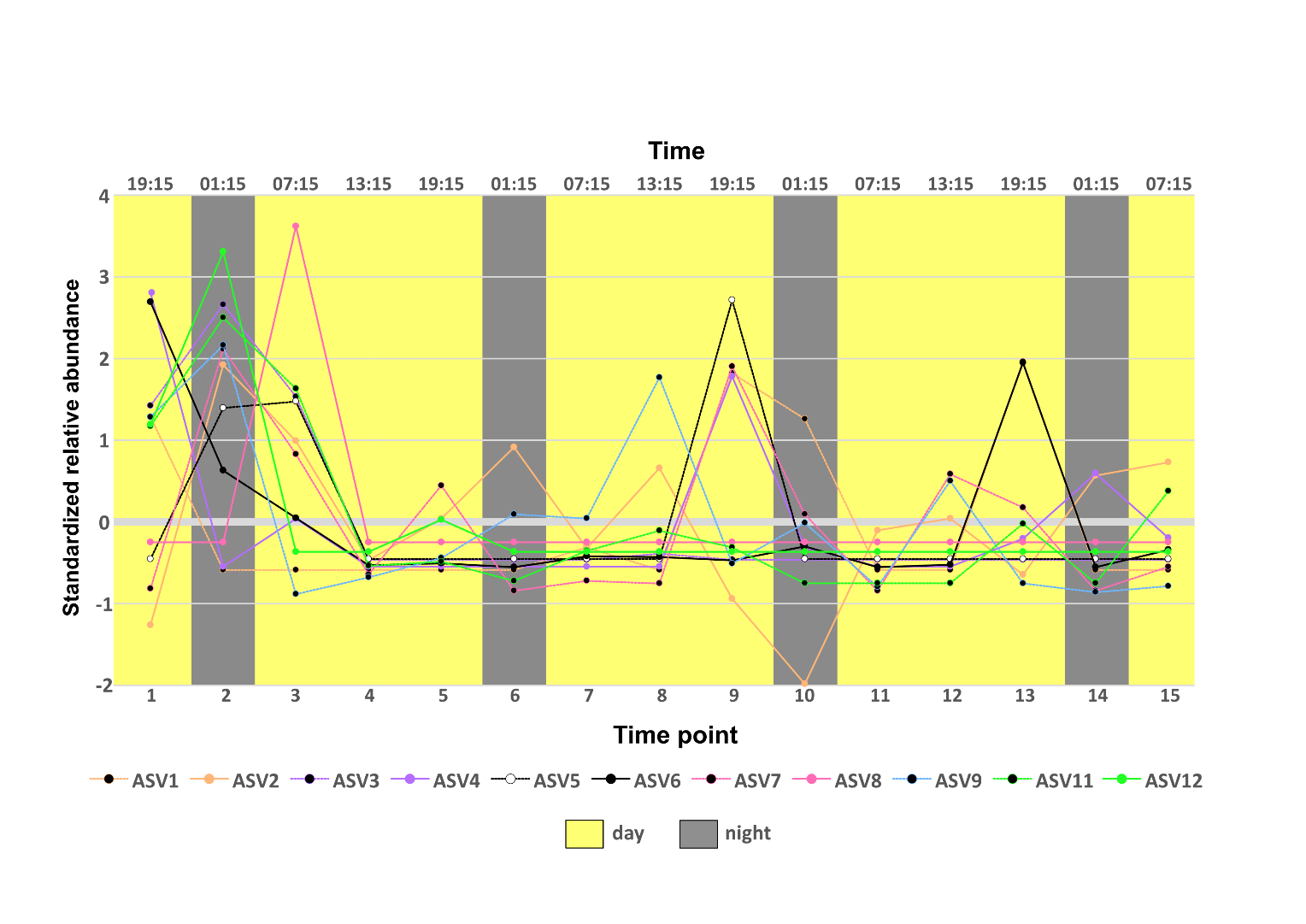


**Figure S15.** Relative abundance, standardized as the z-score, across the sampling time in June for the early riser peat bog ASVs that exhibited a diel cyclic pattern in September. ASV10 was not detected in June.

*Beta diversity*


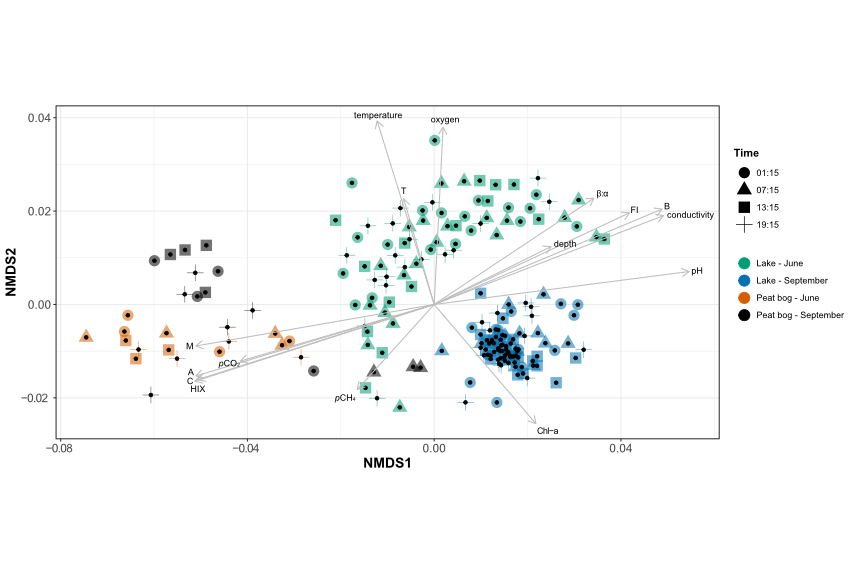


**Figure S16.** NMDS plot (ASV level, stress = 0.196) of bacterial communities in the peat bog and lake Klocka, calculated using a Bray-Curtis dissimilarity matrix, for the sampling periods in June and September. Symbols represent individual samples (189 in total), they are colored according to the combination of site and month and they are shaped based on sampling time. Arrows represent significant (p < 0.001) correlations of environmental variables with bacterioplankton communities. The arrows length is proportional to the correlation between ordination and environmental variable. “A”, “C”, “M”, “T” and “B” stand for peaks A, C, M, T and B respectively. “FI” stands for the fluorescence index, “HIX” for the humification index and “β:α” for the freshness index. Lastly, “pCO_2_” and “pCH_4_” stand for the partial pressure of carbon dioxide and methane respectively.

*Beta diversity*

**Table S3.** Analysis of Bray-Curtis dissimilarities among all communities and per site. PERMANOVAs were performed with *adonis2* function and differences in dispersion were measured using the *betadisper* function from the vegan package in R, followed by ANOVA tests. We also report stress values for NMDS plots. Significant p-values are in bold characters.

| **Group** | **Number of ASVs** | **Factor** | **PERMANOVA (*adonis2*)** | | | **Dispersion (*betadisper*)** | | | **NMDS stress** |
| --- | --- | --- | --- | --- | --- | --- | --- | --- | --- |
|  |  |  | F value | R^2^ | p value | df | F value | p value |  |
| All communities | 52 285 | site | 3.36 | 0.02 | **0.001** | 1,187 | 30.74 | **<0.001** | 0.196 |
|  |  | month | 3.60 | 0.02 | **0.001** | 1, 187 | 40.44 | **<0.001** |  |
|  |  | site- month interaction | 1.76 | 0.01 | **0.001** |  | | |  |
| Lake Klocka | 38 321 | month | 3.95 | 0.02 | **0.001** | 1,156 | 97.13 | **<0.001** | 0.268 |
|  |  | time | 0.99 | 0.02 | 0.989 | 3, 154 | 0.15 | 0.932 |  |
|  |  | depth | 0.99 | 0.02 | 0.994 | 4, 153 | 0.03 | 0.998 |  |
| Peat bog | 15 521 | month | 1.49 | 0.05 | **0.001** | 1, 29 | 8.19 | **<0.01** | 0.160 |
|  |  | time | 1.02 | 0.10 | 0.201 | 3, 27 | 1.81 | 0.168 |  |
| Lake Klocka  (June) | 21 854 | time | 0.98 | 0.04 | 0.80 | 3, 69 | 3.08 | **0.03** | 0.326 |
|  |  | depth | 0.99 | 0.06 | 0.66 | 4, 68 | 0.39 | 0.82 |  |
|  |  | time- depth interaction | 0.99 | 0.17 | 0.79 |  | | |  |
| Lake Klocka  (September) | 20 128 | time | 1 | 0.04 | 0.52 | 3, 81 | 0.87 | 0.46 | 0.253 |
|  |  | depth | 0.98 | 0.05 | 0.73 | 4, 80 | 0.05 | 1 |  |
|  |  | time- depth interaction | 0.99 | 0.14 | 0.68 |  | | |  |
| Peat bog (June) | 11 034 | time | 0.98 | 0.21 | 0.76 | 3, 11 | 33.42 | **<0.001** | 0.150 |
| Peat bog (September) | 6 752 | time | 1.06 | 0.21 | **0.04** | 3, 12 | 6.31 | **<0.01** | 0.138 |
